# Multiple weak brakes act in concert to control STIM1 and store-operated calcium entry

**DOI:** 10.1073/pnas.2518622122

**Published:** 2025-12-08

**Authors:** Ruoyi Qiu, Richard S. Lewis

**Affiliations:** ^a^Department of Molecular and Cellular Physiology, Stanford University School of Medicine, Stanford, CA 94305

**Keywords:** calcium signaling, single-molecule FRET, store-operated calcium entry, STIM1

## Abstract

Store-operated Ca^2+^ entry is a key signaling pathway controlled by the interaction of the ER Ca^2+^ sensor STIM1 with the Orai1 Ca^2+^ channel following ER Ca^2+^ depletion. To avoid generating pathological effects, STIM1 must remain mostly inactive under resting, ER-replete conditions yet respond rapidly and reversibly to changes in ER Ca^2+^ content. It is not well understood how these conflicting requirements are met. Here we combine single-molecule FRET measurements of full-length dimeric STIM1 in lipid membranes with an AlphaFold2 structural model to describe the structure and regulation of the resting state. We show that STIM1 activity is controlled by the combined operation of four relatively weak restraints, or brakes. The Ca^2+^-bound EF-SAM luminal domain acts as a steric restraint to inhibit spontaneous activity. In the cytosolic region, the domain-swapped hydrophobic interaction and alignment of CC1α1 with CC3 of the CRAC activation domain (CAD) positions the apex of CAD next to the ER membrane, where electrostatic lipid–protein interactions further stabilize the inactive conformation. A fourth brake is created by hydrophobic and electrostatic interactions of the two CC1α2/3 domains attached to the base of CAD. Disruption of any one of these brakes triggers spontaneous STIM1 activation, showing that the concerted action of these relatively weak restraints serves to minimize spontaneous activity in resting cells with full ER Ca^2+^ stores, while allowing rapid activation in response to changes in store content.

## Significance

Store-operated calcium entry (SOCE) is essential for many physiological processes, and inadequate regulation of this pathway leads to a variety of human diseases. The ER calcium-sensing protein STIM1 is known to control SOCE, but a structural understanding of STIM1 regulation has been precluded by the protein’s high flexibility. Here we apply single-molecule FRET measurements and structural modeling to identify four weak restraints, or brakes, all of which must operate together to stabilize the inactive state of STIM1 in resting cells. Our findings explain how STIM1 is reliably kept silent under resting conditions, while preserving the critical capacity for rapid and reversible activation in stimulated cells.

Store-operated Ca^2+^ entry (SOCE) is a nearly ubiquitous Ca^2+^ entry pathway activated by cell-surface receptors that release ER Ca^2+^, typically through the activation of IP_3_ receptors in the endoplasmic reticulum (ER) (1). SOCE is essential for the immune response, as well as proper muscle development and function, exocrine secretion, and many other functions, and naturally occurring mutations have been linked to a spectrum of human health disorders (2–5). Gain-of-function (GOF) mutations lead to tubular aggregate myopathy, Stormorken syndrome, and York platelet syndrome, while loss-of-function (LOF) mutations cause immunodeficiency, muscular hypotonia and atrophy, and autoimmunity, among other disorders, demonstrating the critical importance of regulation and reversibility of SOCE (6, 7).

**Fig. 1.**
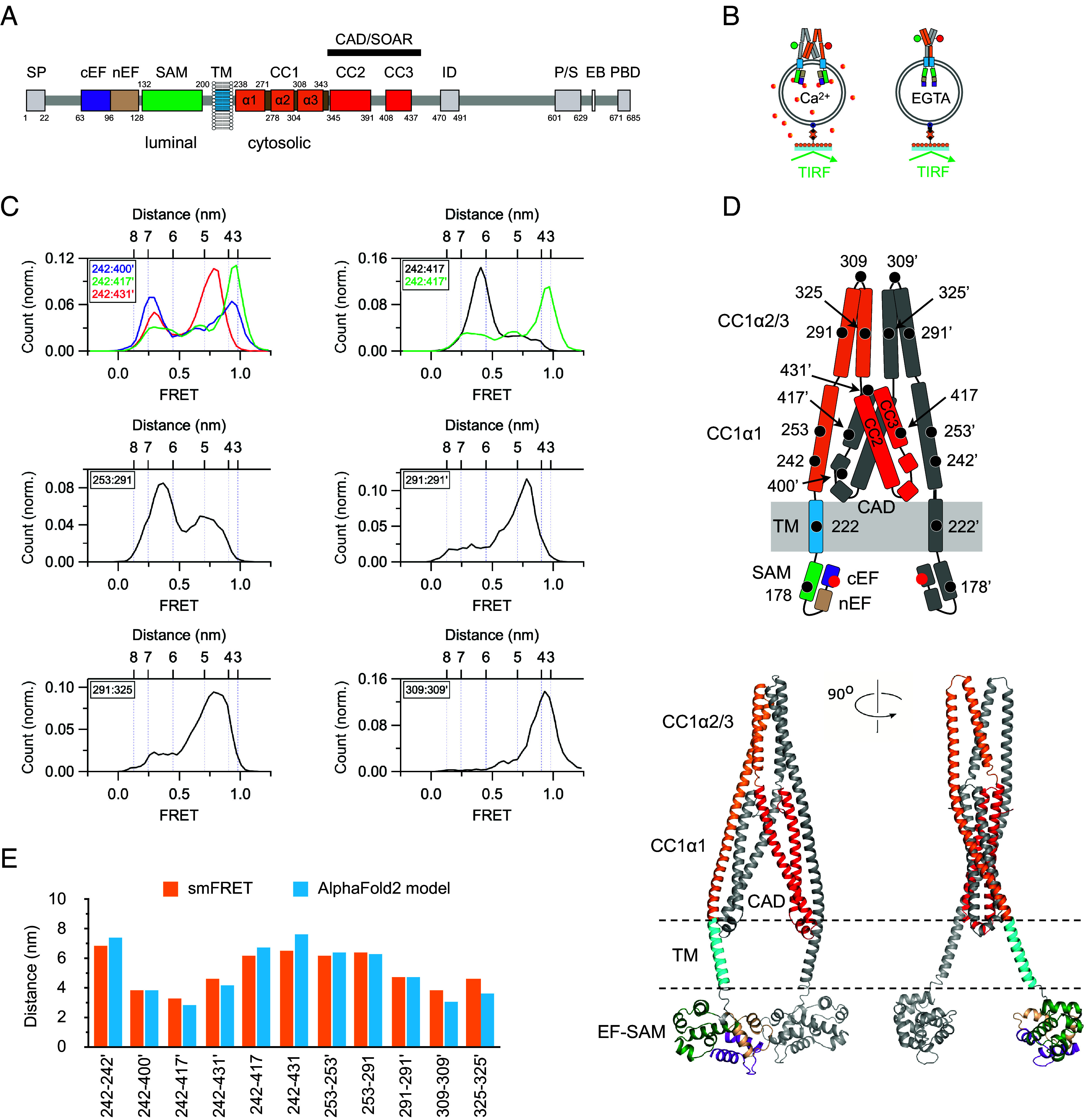
Conformation of STIM1 in the Ca^2+^-bound inactive state. (*A*) Diagram of STIM1 functional domains: SP, signal peptide; cEF, canonical EF hand; nEF, noncanonical EF hand; SAM, sterile alpha motif; TM, transmembrane domain; CC1-3, coiled-coil 1 to 3; ID, inactivation domain; P/S, proline/serine-rich region; EB, EB1 binding domain; PBD, polybasic domain; CAD, CRAC activation domain; SOAR, STIM-Orai activation region. Residue numbers for each domain are indicated. (*B*) flSTIM1 labeled with donor and acceptor dyes is incorporated into liposomes in the presence of 2 mM Ca^2+^ or 0.5 mM EGTA and attached to a coverglass substrate via a biotin–streptavidin linker. The donor dye is excited in TIRF mode, and donor and acceptor dye emissions are used to calculate FRET. (*C*) Selected smFRET amplitude histograms in 2 mM Ca^2+^ supporting key features of the structure in *D*. 242:400’ (n = 188 molecules), 242:417’ (n = 190), 242:431’ (n = 189), 242:417 (n = 291), 253:291 (n = 254), 291:291’ (n = 185), 291:325 (n = 202), 309:309’ (n = 76). Distances are calculated from FRET values using an R_0_ value of 5.8 nm. (*D*) Diagram of STIM1 topology (*Top*) and an AlphaFold2 model (*Bottom*) consistent with smFRET measurements. For clarity, both show only the structure from the cEF hand to the N-terminal end of CAD (residues 63 to 444). Colors correspond to the domain diagram in *A*, and bound Ca^2+^ ions are indicated by the red dots. (*E*) A comparison of interdye distances within the cytosolic domain derived from smFRET measurements and the AlphaFold2 model.

The ER protein STIM1 is the principal regulator of SOCE and operates by controlling access to two critical cytosolic domains: the polybasic domain and CAD (CRAC activation domain), also known as SOAR (STIM1-Orai activating region) or Ccb9 (8–10) (Fig. 1*A*). Depletion of ER Ca^2+^ is sensed by the luminal cEF hand of STIM1, triggering a large conformational change that 1) exposes the polybasic domain to bind phosphoinositide plasma membrane (PM) lipids and promote accumulation at ER–PM junctions, and 2) extends the CAD toward the PM where it binds and activates the CRAC channel Orai1. STIM1 acts as a switch: once activated, all subsequent steps leading to SOCE occur through a passive diffusion trap mechanism, and the sequence is reversed upon refilling of ER Ca^2+^ stores (1).

To avoid GOF pathologies such as Stormorken syndrome and tubular aggregate myopathy, STIM1 must be inactive at resting levels of ER Ca^2+^ (~0.5 mM). Multiple lines of evidence support a critical role of the CC1α1 domain and the CC3 domain of CAD in enforcing the inactive state, often referred to as the “CC1 clamp.” The isolated CC1α1 and CC3 domains interact directly as shown by FRET (11) and a split-STIM assay in which a STIM1 construct truncated at the C-terminal end of CC1α1 traps a soluble cytosolic CAD fragment and releases it upon ER Ca^2+^ depletion (12–16). While multiple mutations in CC1α1 and CC3 are known to inhibit their physical interaction and produce constitutive ([Ca^2+^]_ER_-independent) SOCE (14, 17, 18), the actual binding interface has not been identified. The CC1α3 domain has also been proposed to help maintain the inactive state based on mutagenesis or deletion, but its potential binding partners and underlying mechanisms remain unclear (19, 20). A widely held view is that the CC1 clamp maintains STIM1 in an inactive conformation when the ER Ca^2+^ store is full; Ca^2+^ depletion promotes dimerization of the luminal SAM domains, initiating release of the clamp, coiled-coil formation by the TM and CC1α1 domains, and extension of CAD to the PM where it engages and activates Orai1 (21, 22). However, questions remain about the number and structural origins of the restraints, or “brakes,” that operate to prevent spontaneous activation of STIM1 in cells under resting conditions, while allowing reversible activation in response to depletion of ER Ca^2+^ content.

Attempts to gain deeper insights into the mechanisms of STIM1 regulation using structural approaches like X-ray crystallography or cryo-EM have thus far been thwarted by the high flexibility of full-length STIM1 (flSTIM1). What structural information we have derives from studies of STIM1 fragments: a crystal structure of CAD (23) and NMR structures of the Ca^2+^-bound EF-SAM domain (24), the CC1 monomer (25), and a CC1α3-CC2 fragment (26). While these partial structures can provide much useful information, it is often difficult to know a priori whether and under what conditions they exist in the full-length protein and if they do, what their functions are. Tests based on the functional effects of STIM1 mutagenesis on SOCE are complicated by the fact that some sites appear to stabilize both inactive and active STIM1 conformations (27, 28) or interactions with Orai1 (26).

Single-molecule Förster resonance energy transfer (smFRET) offers information about intramolecular distances as well as conformational dynamics and is thus particularly well suited for studying flexible proteins like STIM1 (29). To gain insight into the organization of the cytosolic domain in the resting state, we initially applied smFRET to a cytosolic domain fragment of STIM1 (ctSTIM1; aa 233 to 685) (30). We found that in ctSTIM1, CC1α1 associates with CAD in a domain-swapped configuration with an orientation predicted to position the CAD apex, the region thought to bind to Orai1 (31–34), next to the ER membrane, and with CC1α2-CC1α3 hairpin structures directed away from the base of CAD (30). However, while ctSTIM1 is a convenient model for studying the inactive conformation of the cytosolic domain, the absence of the transmembrane and luminal EF hands and SAM domains severely limits its utility for exploring mechanisms of STIM1 regulation. For example, the lack of membrane attachment in ctSTIM1 is likely to alter the forces that act on the cytosolic domain to control CC1–CAD interactions, while the absence of luminal domains precludes studies of how Ca^2+^ unbinding triggers activation.

Here, we report the development and application of a system that allows studies of flSTIM1 embedded in artificial membranes. Using smFRET-derived intramolecular distance mapping, we identified an AlphaFold2 model that reveals the location and basis for multiple brakes on STIM1 activation, including domain-swapped interactions of CC1α1 with CC3, intersubunit interactions between CC1α2 and CC1α3 helices, and electrostatic attraction between the apex of CAD and the membrane in which STIM1 is embedded. Together, these studies illustrate how multiple intramolecular restraints prevent pathophysiological activation of STIM1 and SOCE in resting cells while preserving rapid response kinetics.

## Results

### flSTIM1 Resting State Structure Under Ca^2+^-Saturated Conditions.

To prepare flSTIM1 for dye labeling, we first replaced all of the endogenous cysteines (C49, C56, C227, and C437) with serines. These substitutions did not significantly affect STIM1 activity as indicated by a normal resting [Ca^2+^]_i_ and amplitude of thapsigargin-induced SOCE (*SI Appendix*, Fig. S1 and *Methods*). After introducing new cysteines at selected sites, purified flSTIM1 was labeled with donor and acceptor fluorophores, reconstituted in liposomes in the presence of 2 mM Ca^2+^ or 0.5 mM EGTA, and attached to coverslips for smFRET measurements by TIRF microscopy (Fig. 1*B*). Unless otherwise noted, all data in this study were collected in the presence of 2 mM Ca^2+^, which is expected to saturate the luminal binding sites and generate the inactive structure, given the EF-hand affinity of ~200 µM (35–38).

smFRET values were collected from 15 dye pairs positioned throughout CC1 and CAD, as summarized in normalized amplitude histograms in Fig. 1*C* and *SI Appendix*, Fig. S2. Peak FRET values were converted to distances (*SI Appendix*, Table S1) to constrain a coarse-grained model of the STIM1 resting conformation (Fig. 1*D*), revealing several major features (we use “:” throughout to denote a pair of sites in the dimer and “’” to indicate a site on the adjacent subunit). Distances from 242:400’, 242:417’, and 242:431’ dye pairs indicate that CC1α1 is parallel to CC3 in CAD, and the result that 242 is closer to 417’ than 417 indicates a domain-swapped configuration. The parallel association of CC1α1 with CC3 orients the apex of CAD to face the membrane and keeps the TM and luminal SAM domains well separated, while the domain-swapped configuration adds connections between the two STIM1 subunits, stabilizing the dimeric structure beyond what is provided solely by interactions of the two CC2-CC3 hairpins within the basal region of CAD (23). Multiple smFRET-derived distances suggest that the CC1α2 and CC1α3 domains form hairpin structures (291:325) that are closely associated (291:291’, 309:309’, 325:325’) and point away from the base of CAD (253:291). This arrangement of the cytosolic region and corresponding smFRET values are similar to those of the soluble cytosolic fragment of STIM1 (ctSTIM1) we described in a previous smFRET study (30) (*SI Appendix*, Table S2), although there are important differences in dynamics stemming from the presence of membrane anchors in the flSTIM1 structure (described below).

To gain more insight into the resting structure in saturating Ca^2+^ and the intramolecular contacts that stabilize it, we generated a series of AlphaFold2 models for the flSTIM1 dimer. Out of the top five models (*SI Appendix*, Fig. S3), one mirrored the coarse-grained model and was highly consistent with the smFRET data in the cytosolic region (Fig. 1*D*). Within this region, the interdye distances simulated on the AlphaFold2 model agree closely with distances calculated from smFRET (Fig. 1*E* and *SI Appendix*, Table S1). While the model needs further refinement for the TM and luminal domains (*SI Appendix*, *Methods*), it proved to be quite useful in revealing important interaction sites within the cytosolic domain, as described below.

**Fig. 2.**
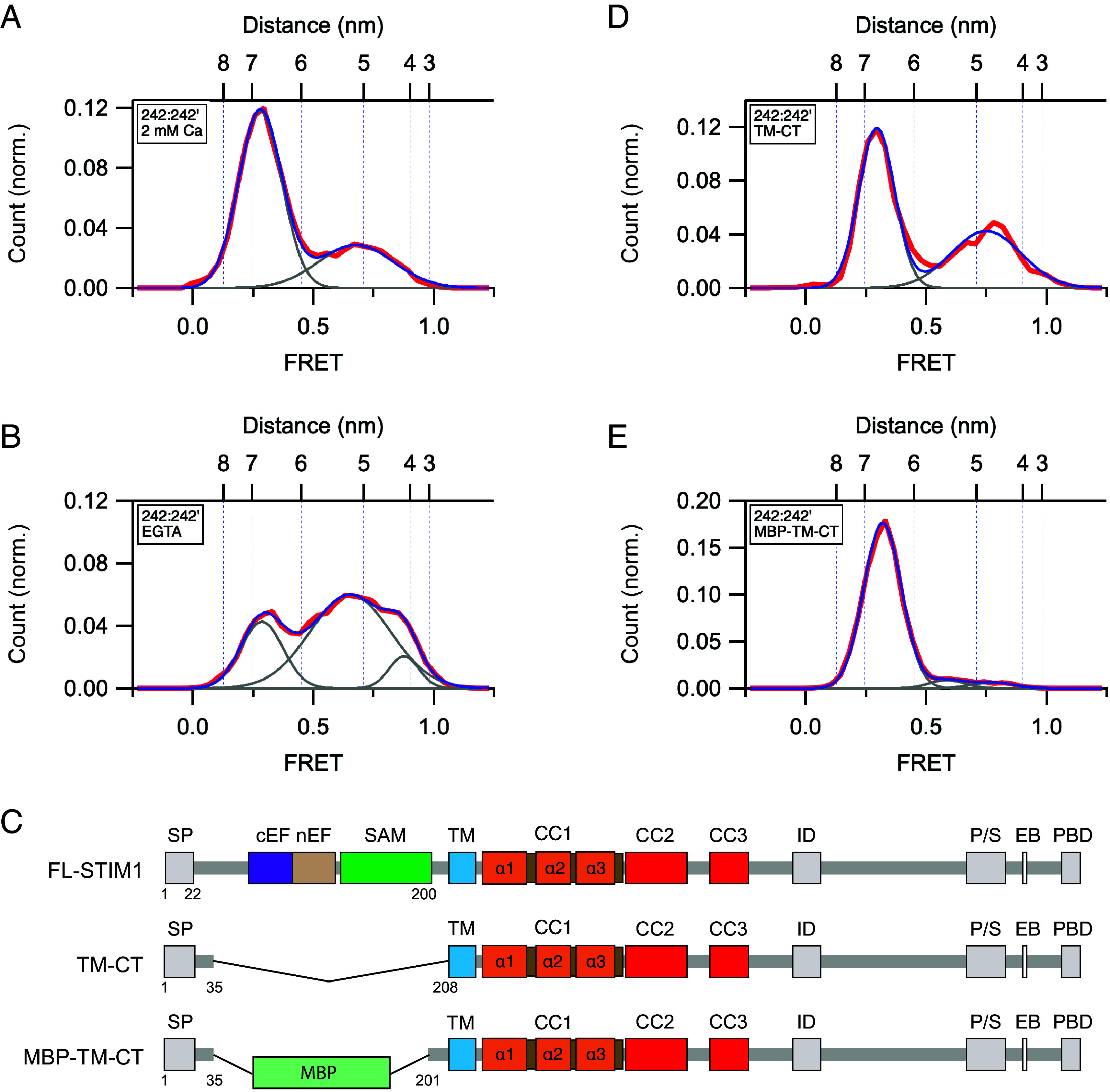
The Ca^2+^-bound EF-SAM domain inhibits spontaneous STIM1 activity through a steric mechanism. (*A*) smFRET histogram for the 242:242’ dye pair in 2 mM Ca^2+^ (n = 361). Fitted Gaussian curves (gray) and their sum (blue) are superimposed on the data (red). Fit parameters (peak FRET and fractional area): 0.28 (70%), 0.68 (30%). (*B*) smFRET histograms for the 242:242’ dye pair in 0.5 mM EGTA (n = 437). Fit parameters (peak FRET and fractional area): 0.29 (25%), 0.65 (67%), 0.88 (8%). In *A* and *B*, the area under the low-FRET peak is used to indicate the probability of the resting conformation. (*C*) Diagrams of full-length STIM1, TM-CT, and MBP-TM-CT constructs. (*D*) smFRET histograms for 242:242’ of STIM1 with deleted luminal domain (TM-CT; n = 203). Fit parameters (peak FRET and fractional area): 0.30 (59%), 0.75 (41%). (*E*) smFRET histograms for STIM1 with MBP substituted for the luminal domain (MBP-TM-CT; n = 234). Fit parameters (peak FRET and fractional area): 0.32 (92%), 0.59 (4%), 0.78 (4%).

The stability of the resting conformation shown in Fig. 1*D* is shown by the large area under the predominant FRET peaks at multiple dye locations (Fig. 1*C*). As a convenient metric of resting state occupancy, we chose the area under the low-FRET (0.28) peak of the 242:242’ pair, because the peak was well separated from higher peaks and its area fell significantly (70% to 25%) in response to EGTA as the distribution shifted to higher FRET states (Fig. 2 *A* and *B*). Below, we quantify the 242:242’ low-FRET probability to assess the function of intramolecular brakes that stabilize the resting state under conditions of saturating Ca^2+^.

### The Ca^2+^-Bound EF-SAM Domain is a Steric Brake.

Following store depletion, release of the EF hands from the SAM domains is well recognized as the critical initiating step in STIM1 activation by allowing SAM–SAM dimerization (24, 39). A previous report that overexpression of an EF-SAM deletion mutant of STIM1 evoked spontaneous SOCE (40) led us to hypothesize that the Ca^2+^-bound EF-SAM domain may also act as a steric brake on spontaneous activation. To test this using smFRET, we assessed changes in the resting state probability after removing the EF-SAM domain and after replacing it with maltose binding protein (MBP), a well-folded, monomeric protein (Fig. 2*C*). The probability of the resting state was measured from the relative area under the low-FRET peak from smFRET measurements of the 242:242’ dye pair. In the presence of saturating Ca^2+^, removal of the EF-SAM domain reduced occupancy of the low-FRET state from 70% to 59% and increased the proportion of molecules with higher FRET, indicating destabilization of the resting state (Fig. 2*D*). Replacing the EF-SAM domain with MBP reversed this effect and even enhanced the resting state occupancy beyond normal (91%; Fig. 2*E*), probably due to its larger size of 43 kD, compared to 16 kD for the EF-SAM domain. These results offer quantitative evidence that the Ca^2+^-bound EF-SAM domains create a brake on STIM1 activation by acting as a steric barrier that hinders the close apposition of the luminal and presumably the TM and CC1α1 domains, inhibiting formation of the extended coiled-coil and the active state (28).

### Alignment of the CC1α1–CC3 Interface Positions the CAD Apex at the ER Membrane.

The binding of CC1α1 to CC3 in CAD acts in two ways as a brake. In addition to keeping the CC1α1, TM, and luminal SAM domains well separated to reduce chances for spontaneous dimerization, it also positions the CAD apex, the region implicated in Orai1 activation (31–34), as far as possible from the PM (Fig. 1). While multiple CC1α1 and CC3 residues that enforce the resting state have been identified through mutagenesis (12, 14, 17, 18, 27), the registration of the interhelical interface is unknown. Both helices have hydrophobic faces which could potentially align in a number of ways, and their registration is important in part because it determines the position of the CAD apex relative to the ER membrane.

**Fig. 3.**
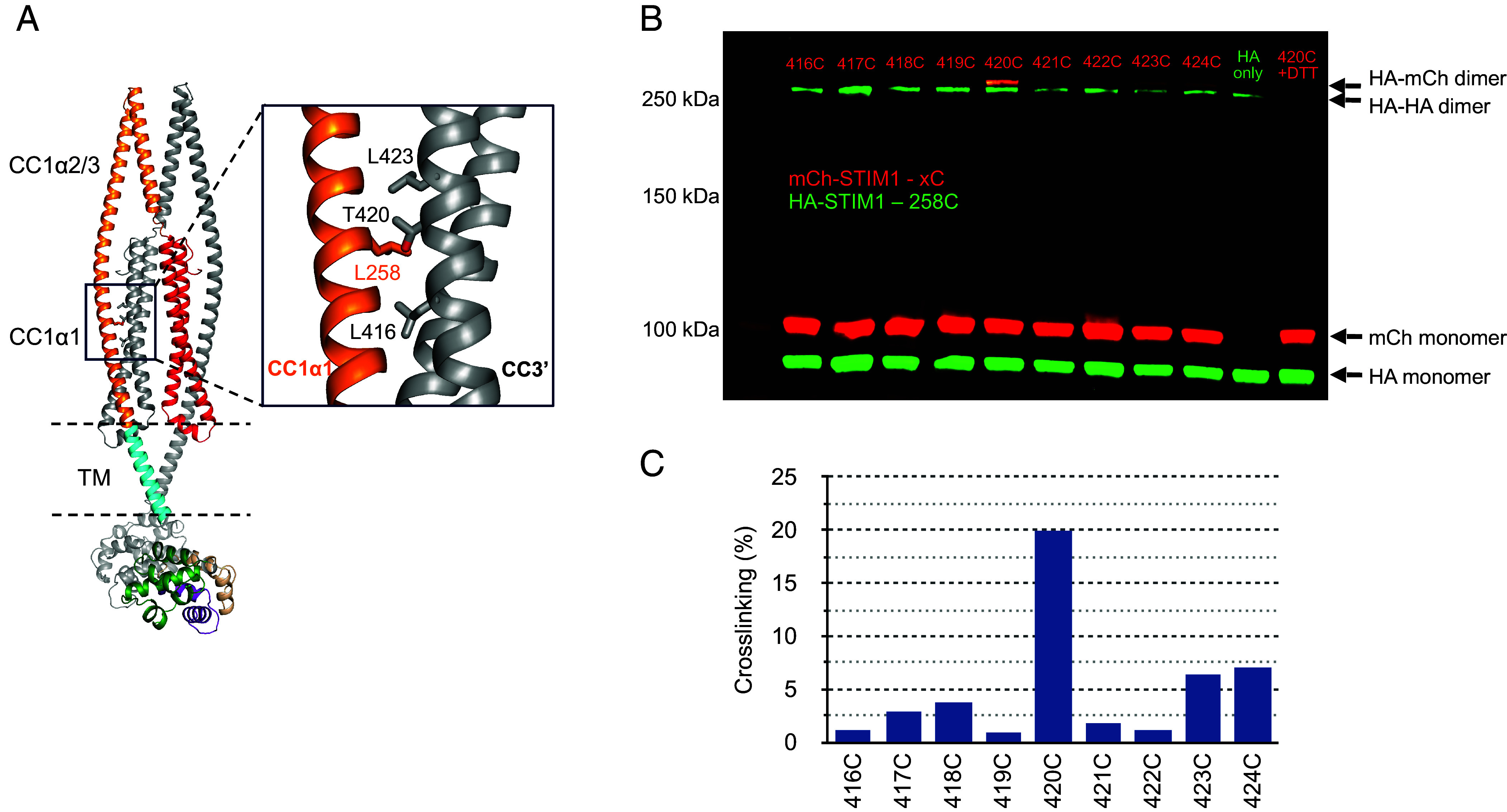
Helical alignment of the CC1α1–CC3 brake. (*A*) Proximity of L258 (CC1α1) and T420 (CC3’) predicted by the AlphaFold2 model. (*B*) Western blot showing heterodimer disulfide crosslinking between HA-STIM1-L258C and mCh-STIM1-T420C. Each lane contains the lysate from diamide-treated cells coexpressing HA-STIM1-L258C (green) and a single mCh-STIM1 mutant (red) (*Methods*). Treatment of the lysate with DTT removes the dimer bands (“420C+DTT”), confirming disulfide crosslinking. Expression of HA-STIM1-L258C alone yields homodimers (“HA only”); this homodimer band is also seen in cells coexpressing HA-STIM1-L258C with mCh-STIM1 mutants. (*C*) Percent of heterodimers forming disulfide crosslinks, measured from the gel in *B* (*Methods*). Results are representative of two experiments.

The AlphaFold2 model predicts close proximity of L258 with T420’, and of L261 with T420’ and L423’ (Fig. 3*A*). We tested these predictions using a cysteine-scanning approach, in which we introduced cysteines at various locations in mCh-STIM1 and coexpressed it in STIM1/2 DKO HEK293 cells with HA-STIM1 bearing the L258C or L261C mutation. Due to the domain-swapped interactions of CC1α1 and CC3’, close proximity of the introduced cysteines would be detectable by the formation of disulfide-linked heterodimers. Following treatment with the membrane-permeant oxidizing agent diamide, disulfide-linked dimers were quantified by Western blot (*Methods*) (Fig. 3 *B* and *C* and *SI Appendix*, Fig. S4). L258C and L261C by themselves form some homodimers after diamide treatment. This may be explained by a weakening of the CC1α1–CC3 clamp which has been observed following substitutions of smaller, less hydrophobic residues (ser, gly, or ala) at these sites (12, 14, 18), potentially allowing transitions to an active CC1α1 coiled-coil state that permit disulfide bonds to accumulate over time. Importantly, quantitation of the disulfide heterodimer bands indicated that in the resting state, L258C is most proximal to T420C’ (Fig. 3*C*), while L261C is closest to T420C’ and L423C’ (*SI Appendix*, Fig. S4*C*). The formation of multiple disulfides is consistent with the AlphaFold2 structure and suggests some flexibility of the side chains. Together, these results support the ability of the AlphaFold2 model to describe the clamp interface and establish the alignment of the CC1α1 and CC3 helices in the resting state.

**Fig. 4.**
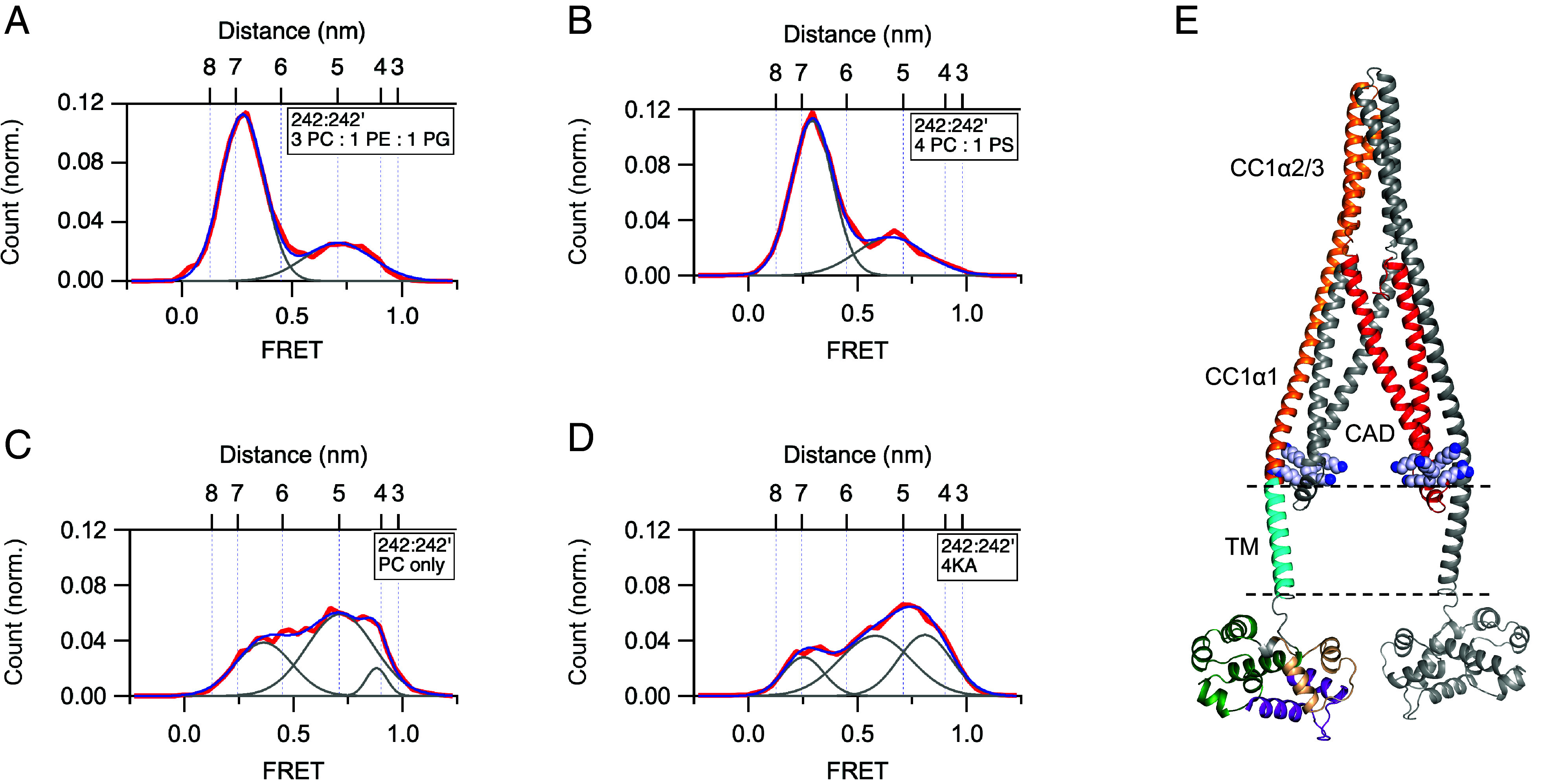
Electrostatic interaction of the CAD apex with the liposome membrane enhances the stability of the STIM1 resting state. flSTIM1 was reconstituted in the presence of 2 mM Ca^2+^ in liposomes of varying composition and smFRET histograms were collected for 242:242’. Fitted Gaussian curves are superimposed to measure occupancy of the low-FRET state. (*A*) Liposome composition 3 PC:1 PE:1 PG (20% negative charge; n = 361). The histogram is reproduced from Fig. 2*A*. Fit parameters (peak FRET and fractional area): 0.28 (70%), 0.68 (30%). (*B*) Liposome composition 4 PC:1 PS (20% negative charge; n = 204). Fit parameters (peak FRET and fractional area): 0.29 (71%), 0.65 (29%). (*C*) Liposome composition PC only (uncharged; n = 184). The lack of negatively charged lipids destabilizes the resting state. Fit parameters (peak FRET and fractional area): 0.36 (33%), 0.72 (60%), 0.88 (6%). (*D*) STIM1-4KA (K382A/K384A/K385A/K386A) destabilizes the resting state when reconstituted in negatively charged liposomes containing 3 PC:1 PE:1 PG (20% negative charge; n = 243). Fit parameters (peak FRET and fractional area): 0.25 (17%), 0.58 (47%), 0.81 (36%). (*E*) AlphaFold2 structure highlighting the position of the KIKKKR domain adjacent to the liposome membrane. Lysines and arginines are shown as spheres.

Interestingly, the registration between CC1α1 and CC3 predicts a close apposition of the CAD apex with the ER membrane, raising the possibility that protein–lipid interactions may influence the stability of the resting state. To address this, we examined the effects of lipid charge on the resting conformation probability in saturating Ca^2+^ as judged by smFRET of the 242:242’ dye pair. With liposomes made from 80% phosphatidylcholine (PC; neutral) + 20% phosphatidylserine (PS; one net negative charge), probability of the low-FRET state was 71%, similar to the 70% observed with our standard lipid mixture having the same net charge [60% PC + 20% phosphatidylethanolamine (PE, neutral) + 20% phosphatidylglycerol (PG, one net negative charge)] (Fig. 4 *A* and *B*). In contrast, in liposomes containing only neutral PC, the probability of the low-FRET state was reduced to 33% (Fig. 4*C*). Similar results were obtained with neutral liposomes containing 80% PC and 20% PE (*SI Appendix*, Fig. S5*A*). These results demonstrate that negatively charged lipid headgroups serve as a restraint that helps stabilize the resting conformation of STIM1. A potential mechanism for this effect could involve the interaction of negatively charged headgroups with two clusters of basic residues (_382_KIKKKR) located near the apex of CAD (Fig. 4*E*). To test this idea, we mutated the four lysines to alanines (4KA) or glutamines (4KQ) and repeated the smFRET measurements in standard liposomes (3 PC:1 PE:1 PG). The 4KA mutation reduced the probability of the low-FRET resting conformation to 17% (Fig. 4*D*), while 4KQ reduced it to 18% (*SI Appendix*, Fig. S5*B*), supporting the conclusion that an electrostatic protein–lipid brake helps stabilize the resting state.

### The CC1α2/3 Dimer Forms a Fourth Brake on STIM1 Activity.

Studies showing that a quadruple mutation (E318A/E319A/E320A/E322A, or “4EA”) in CC1α3 partially activates STIM1 and SOCE (18–20, 27) suggest a role for CC1α3 in stabilizing the resting state, but an underlying mechanism has not been identified. We hypothesized that the intersubunit association of the CC1α2 and CC1α3 helices (Fig. 1) forms a brake on STIM1 activation, and that the 4EA mutation may activate STIM1 by destabilizing this structure. As shown in *SI Appendix*, Fig. S6*A*, 4EA promoted separation of the CC1α2/3 dimer in saturating Ca^2+^, as indicated by reduced occupancy of the 309:309’ high-FRET state. In addition, as shown by 242:242’ FRET, the 4EA mutation released CC1α1 from CAD in the presence of Ca^2+^ to a similar extent as EGTA did with WT STIM1 (cf. Fig. 2*B* and *SI Appendix*, Fig. S6*B*). Consistent with previous studies, STIM1-4EA evoked constitutive SOCE in unstimulated cells (*SI Appendix*, Fig. S6*C*).

**Fig. 5.**
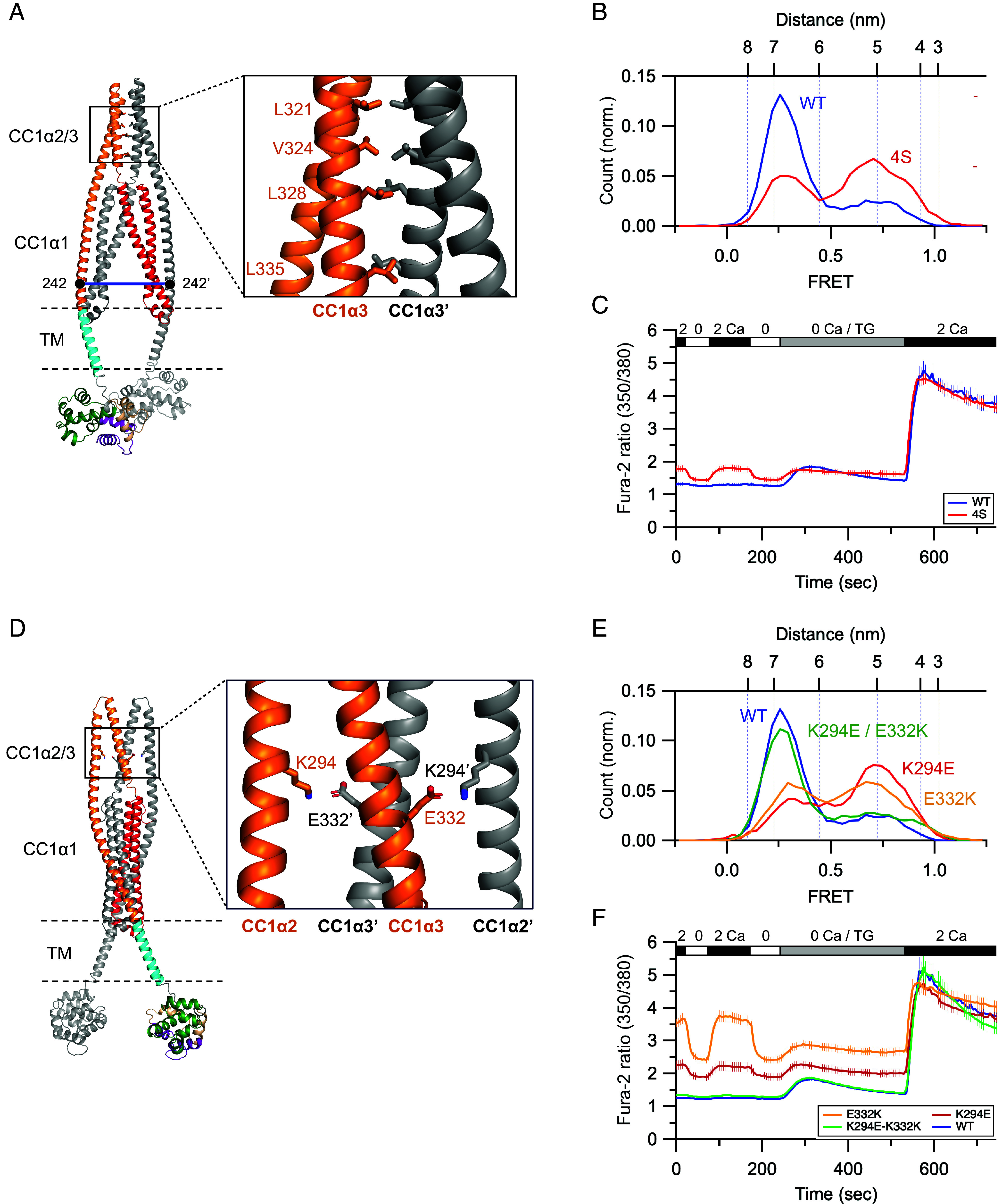
Structural basis of the CC1α2/3 brake. (*A*) A symmetric hydrophobic interface between neighboring CC1α3 domains in the AlphaFold2 model. (*B*) smFRET histogram of 242:242’ in STIM1-WT (from Fig. 2*A*) and STIM1-4S (L321S/V324S/L328S/L335S) in 2 mM Ca^2+^ (n = 218). (*C*) Fura-2 350/380 ratios (mean ± SEM) from resting HEK cells expressing Orai1 and either STIM1-WT (n = 57 cells) or STIM1-4S (n = 63 cells). Changes in extracellular [Ca^2+^] (0 and 2 mM) and addition of thapsigargin (TG; 1 µM) are indicated. Resting [Ca^2+^]_i_ is elevated in cells expressing STIM1-4S, indicating constitutive activity of STIM1 and SOCE, while TG-induced SOCE is normal. (*D*) Intersubunit salt bridges between K294 (CC1α2) and E332 (CC1α3) in the AlphaFold2 model. (*E*) smFRET histogram of 242:242’ in STIM1-WT (from Fig. 2*A*) and STIM1-K294E (n = 211), STIM1-E332K (n = 216), or STIM1-K294E/E332K (n = 255) in 2 mM Ca^2+^. (*F*) Fura-2 350/380 ratios (mean ± SEM) from resting HEK cells expressing Orai1 and either STIM1-WT (from *C*, n = 57 cells), STIM1-K294E (n = 62 cells), STIM1-E332K (n = 87 cells), or STIM1-K294E/E332K (n = 56 cells). Changes in extracellular [Ca^2+^] (0 and 2 mM) and addition of thapsigargin (TG; 1 µM) are indicated. Resting [Ca^2+^]_i_ is elevated in cells expressing STIM1-K294E or E332K, indicating constitutive activity of STIM1 and SOCE, but not in cells expressing the double mutant K294E/E332K. In all cases, TG-induced SOCE is normal.

To gain structural insight into the basis of CC1α2/3 as a brake, we used the AlphaFold2 model to identify residues that could stabilize the CC1α2/3 dimer in the resting state. The model predicts homotypic hydrophobic interactions between the two CC1α3 subunits at four locations: L321, V324, L328, and L335, as well as symmetric intersubunit salt bridges between CC1α2 and CC1α3’ at K294 and E332 (Fig. 5 *A* and *D*). Mutating the four hydrophobic sites to serines (“4S” mutant) significantly destabilized the resting state as assayed by 242:242’ FRET (Fig. 5*B*). When expressed with Orai1 in HEK cells, the 4S mutant STIM1 evoked constitutive Ca^2+^ influx (Fig. 5*C*). The single mutations K294E or E332K produced an even stronger activating effect as judged by destabilization of the resting state in smFRET measurements (Fig. 5*E*) and stimulation of constitutive Ca^2+^ entry in cells (Fig. 5*F*). Importantly, the charge-swapped double mutant K294E/E332K returned STIM1 to its resting state as indicated by 242:242’ smFRET and restoration of normal resting [Ca^2+^]_i_ in cells (Fig. 5 *E* and *F*), strongly supporting a direct interaction between the two sidechains. Together, these data reveal specific hydrophobic and electrostatic interactions that stabilize the CC1α2/3 dimer in the resting state to create a brake that minimizes spontaneous STIM1 activation.

### Membrane Insertion of flSTIM1 Affects Conformational Dynamics of the Resting State.

**Fig. 6.**
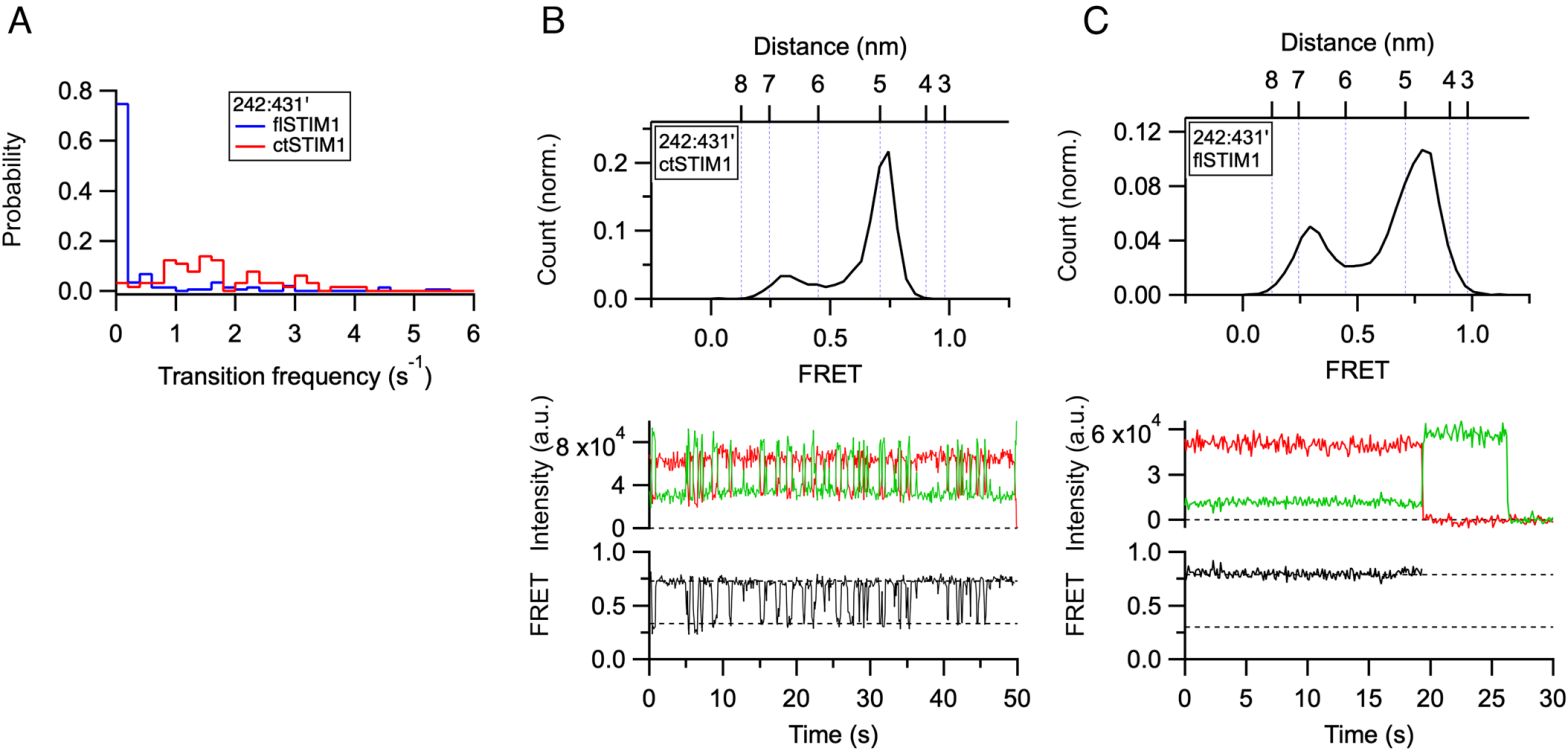
Membrane insertion of flSTIM1 reduces conformational fluctuations out of the resting state. (*A*) Probability distribution of the mean smFRET transition frequency over a 5-s period for flSTIM1 and ctSTIM1, measured for the 242:431’ dye pair (CC1α1–CC3’); n = 189 (flSTIM1) and n = 78 (ctSTIM1). (*B*) smFRET histogram for ctSTIM1 242:431’ (*Top*), and representative traces (*Bottom*) of donor (green) and acceptor (red) fluorescence and FRET (black). Dashed lines indicate peak values from the smFRET histogram. ctSTIM1 makes frequent transitions out of the high-FRET resting state due to switching between domain-swapped and nonswapped conformations (see text). (*C*) smFRET histogram for flSTIM1 242:431’ (*Top*; from Fig. 1*C*), and representative traces (*Bottom*) of donor (green) and acceptor (red) fluorescence and FRET (black). Acceptor photobleaching followed by donor photobleaching is shown. Dashed lines indicate peak values from the smFRET histogram. Note the long stable occupancy of the high-FRET resting state.

The similarity of key smFRET values in flSTIM1 and ctSTIM1 (*SI Appendix*, Table S2) shows that membrane insertion of the TM domains in flSTIM1 does not significantly affect the gross structure of the cytosolic domain; however, it does create significant differences in conformational dynamics. In ctSTIM1, we previously observed frequent switching between domain-swapped and nonswapped conformations of the CC1α1–CC3 clamp (30). To compare clamp switching in ctSTIM1 and flSTIM1, we counted FRET transitions for the 242:431’ dye pair occurring within the first 5 s of single-molecule traces. While ctSTIM1 displayed frequent transitions, flSTIM1 only rarely fluctuated, with molecules showing constant FRET values for up to tens of seconds (Fig. 6). This increased stability of the domain-swapped conformation may result from membrane insertion as well as electrostatic interaction of lipid headgroups with the CAD apex (Fig. 4), and it highlights the advantages of membrane-reconstituted flSTIM1 as a model system for identifying the physical interactions that govern STIM1 activity.

## Discussion

In this study, we combined smFRET, cysteine crosslinking, and AlphaFold2 modeling to describe the structure of full-length STIM1 in a membrane environment in the Ca^2+^-bound state. The model proved to be accurate enough to reveal functionally important interactions of sidechains in multiple critical regions that control STIM1 activity.

### Multiple Weak Brakes Control STIM1 Activation.

We found that the stability of the inactive Ca^2+^-bound state is determined by at least four weak brakes discussed below, all of which are required to minimize spontaneous activation of STIM1.

*EF-SAM domain steric brake*: Several reports have described spontaneous puncta formation and SOCE in cells overexpressing a truncated STIM1 variant lacking the EF-SAM domain, suggesting that the Ca^2+^-bound luminal domain itself may suppress spontaneous activity (16, 18, 40). Our results confirm these findings and suggest a steric mechanism in which the bulk of the Ca^2+^-bound EF-SAM domains in STIM1 prevents close approach of the TM and CC1α1 domains, thus limiting the opportunity for dimerization of the TM and CC1 domains and spontaneous activation. Deletion of the EF-SAM domain caused only modest activation compared to the effect of Ca^2+^ removal from the WT protein (cf. Fig. 2 *B* and *D*), highlighting the additional action of SAM–SAM dimerization in promoting and stabilizing the active state. Thus, the luminal domain performs two opposing functions: in the Ca^2+^-bound state in resting cells it acts as a weak brake to help keep STIM1 inactive, while in the Ca^2+^-free state after ER Ca^2+^ depletion it becomes an “accelerator” to promote STIM1 activation through SAM–SAM dimerization. Interestingly, the SAM–SAM FRET measured with a 178:178’ dye pair fluctuated over a wide range and transiently reached high values (*SI Appendix*, Fig. S7), suggesting that even under Ca^2+^-saturated resting conditions, the two domains collide. Such random collisions between SAM domains may be essential for enabling SAM–SAM dimerization and consequent STIM1 activation in response to store depletion. Further studies will address the role of these and other conformational dynamics in promoting the transition from the inactive to the active state.

*CC1α1–CC3 clamp:* Pioneering studies by Romanin, Hogan, Zhou, and their colleagues established the importance of a CC1α1–CC3 interaction in limiting spontaneous activation of STIM1 (11–14, 17, 18). Our model clarifies the structure of the clamp and reveals several mechanisms by which it acts to inhibit activation. First, it shows directly that parallel binding of CC1α1 binding to CC3 keeps both the TMs and the luminal domains well separated in the resting state (Fig. 1), limiting the opportunities for interaction that could lead to spontaneous activation. This contrasts with an earlier model in which the TMs are associated in the resting state and activation occurs through rotation of crossed helices (12, 15). We believe instead that activation involves a significant translational movement of the separated TM domains to bring SAM domains into contact for dimerization. One possible reason for the discrepancy is that TM-CC1 fragments were used in the previous study, and CC1 helices by themselves can bind weakly to each other in the absence of the V-shaped CAD (41) (see ref. 28 for additional discussion).

A second essential aspect of the CC1α1–CC3’ brake is that the parallel arrangement of the two helices points the CAD apex toward the ER membrane. This configuration effectively distances the apex, which is thought to interact with Orai1 to open the channel (31–34), as far as possible from the PM and Orai1 when ER Ca^2+^ stores are full. The register between the helices also sets the proximity of the CAD apex to the ER membrane in the resting state. Multiple computational models of the registration between CC1α1 and CC3 have been proposed based on hypothetical alignments of residues known by mutagenesis to be required for the clamp. These include antiparallel CC1α1 and CC3 helices with L258 paired with V419 (12), as well as parallel helices with L258 paired with L423 (30) or with L416 (42). Using a cysteine scanning approach, we established proximity of residues L258 to T420’ and of L261 to T420’ and L423’, consistent with our AlphaFold2 model (Fig. 3 and *SI Appendix*, Fig. S4). This result differs from all previous models in placing CAD one helical turn further from the membrane than van Dorp et al. (30), one turn closer to the membrane than Horvath et al. (42), and several turns closer than Höglinger et al. (43).

The accurate alignment of CC1α1 and CC3 in our model reveals a hydrophobic interface that may explain many previously reported effects of mutations on STIM1 activation (*SI Appendix*, Fig. S8 and Table S3). The model shows a series of domain-swapped hydrophobic interactions of CC1α1 with CC3: M244 and L248 with I409’, L251 with L416’, L258 with V419’, and L261 and L265 with L423’. Shrestha et al. (14) found that alanine substitutions for L373 (CC2) or L427 (CC3) enhance binding of CAD to CC1 and proposed that the native leucines temper the strength of the clamp. The model shows these leucine side chains in proximity to L251 and L265, respectively, suggesting a possible steric mechanism for this effect. Finally, the model suggests that our previous observations of STIM1 activation by A369K and A376K (41) may result from interference with the hydrophobic domains created by L258’/L416/V419 and L248’/L251’/I409, respectively.

In addition, the model suggests that the CAD apex may extend beyond the edge of the membrane as approximated by the location of Q233. Membrane insertion could help explain why large hydrophobic residues (trp, phe, leu, his) at position 394 in the apex appear to stabilize the inactive state (14, 43, 44), although insertion is not a certainty given the flexibility of the apex (30), and interactions with hydrophobic residues at the base of CC1α1 are also possible (14).

*CAD apex–lipid interaction*: The registration of the CC1α1–CC3 clamp positions the CAD apex close enough to interact with lipid headgroups of the ER membrane. Neutralizing the membrane charge or the two _382_KIKKKR regions destabilized the resting state, supporting the operation of an electrostatic brake (Fig. 4 and *SI Appendix*, Fig. S5). Attraction between the ER membrane and the 10 positive charges of the KIKKKR domains in cells would be expected given the lipid composition of the ER membrane, which contains ~17% negatively charged PS and phosphatidylinositol (45), and physical modeling that predicts a −25 mV isopotential electric field at ~1 nm from a membrane containing 2:1 PC/PS (46). Interestingly, in store-depleted cells where the CAD is extended away from the ER, the KIKKKR domain has been reported to interact with negatively charged PI4P in the PM to help trap STIM1 at ER–PM junctions (47) and to enhance STIM1 binding to Orai1 (19, 32). Thus, like the EF-SAM and CC1 domains, the KIKKKR domain appears to serve two opposing functions: to stabilize the inactive state under resting conditions and to promote the activated state after store depletion.

*CC1α2/3 dimer*: A role for CC1α3 in stabilizing the STIM1 resting state was first proposed by Balla and colleagues, who reported that the 4EA mutation (E318A/E319A/E320A/E322A) in CC1α3 caused partial activation of STIM1 and SOCE (19). Initially, 4EA was thought to disrupt a brake formed by electrostatic interaction of the glutamates with the basic KIKKKR residues near the CAD apex (19), which is not evident in the AlphaFold2 structure of Fig. 1*D*. Later studies ascribed the activating effects instead to disruption of amphiphilic helical properties (20) or stabilization of a potential CC1α3 binding interface for Orai1 (26). Our results with the 4EA mutation support the idea that the amphiphilic CC1α3 helix helps stabilize the CC1α2/3 dimer, which as described below may enhance CC1α1 binding to CC3.

The AlphaFold2 model predicted two sites of interaction that stabilize the close connections between the CC1α2/3 hairpin structures in the resting state: a symmetric hydrophobic interface between CC1α3 domains (L321, V324, L328, L335) and an intersubunit electrostatic interaction between CC1α2 and CC1α3’ (K294<->E332). Mutations at these sites triggered spontaneous STIM1 activation in vitro and constitutive SOCE in cells, identifying the CC1α2/3 dimer as a regulatory domain of STIM1. We propose that the interacting pair of CC1α2/3 hairpin domains stabilizes the interaction of CC1α1 with CAD by holding the CAD in position, so that when CC1α1–CC3 contacts are transiently broken, the CAD preferentially rebinds to CC1α1 rather than escape. This mechanism can explain why in the split STIM1 assay, in which the CC1α3 is not connected directly to CC2 of CAD and therefore cannot hold CAD in position, the CC1α2 and CC1α3 domains fail to enhance CAD trapping beyond what is achieved by the CC1α1 domain alone (12).

### Implications of Multiple Weak Brakes for Regulation of STIM1 and SOCE.

The serious pathological consequences of GOF and LOF mutations in STIM1 and Orai1 underscore the importance of tight STIM1 regulation; it must be strongly suppressed when ER Ca^2+^ stores are replete, yet readily activated by loss of ER Ca^2+^ and deactivated upon store refilling. This study shows that at least four brakes act to restrain the activity of STIM1 under Ca^2+^-saturated conditions, and the effects of mutations indicate that they are all relatively weak. Thus, the release of any one brake by mutating interface residues, e.g., L248S, L251S, and others for the CC1α1–CC3’ clamp (*SI Appendix*, Table S3), _382_KIKKK > AIAAA for the CAD apex–lipid brake (Fig. 4), or K294E or E332K for the CC1α2/3 brake (Fig. 5), triggers significant spontaneous activation, demonstrating that in each case the remaining three brakes together are not sufficient to maintain the inactive state.

The stabilization of the resting state by multiple weak brakes may be understood in terms of avidity, as each brake constrains the degrees of freedom of the others, thereby increasing the probability that the others will engage. At the same time, each brake is intrinsically weak, such that the off rate for each is high enough to optimize the speed of activation upon store depletion. In this way, multiple weak brakes, unlike a single strong one, may ensure reliable suppression of activity in the Ca^2+^-bound state but rapid and reversible activation in response to changes in ER Ca^2+^ store content. These studies are an important step towards understanding the full structural basis for STIM1 stability and reveal multiple intersubunit interaction sites that may ultimately provide pharmacological targets to modulate STIM1 and SOCE for therapeutic purposes in vivo.

## Methods

A brief description of methods is given below; see the *SI Appendix* for full details.

### Cell Culture.

HEK 293 cells were cultured in DMEM containing 2 mM L-alanyl-glutamine, 10% FBS, and 100 U/ml penicillin/streptomycin at 37 °C in 5% CO_2_. Sf9 cells were cultured in ESF 921 insect cell culture medium (Expression Systems) at 27 °C with constant shaking at 120 RPM. Baculovirus was generated with the Bac-to-Bac baculovirus expression system (Invitrogen).

### DNA Constructs.

After mutation of native cysteines in STIM1 (C49, C56, C227, and C437) to serines, desired mutations were introduced by site-directed mutagenesis (Quikchange XL; Stratagene).

### Protein Expression and Purification.

All STIM1 constructs in pFastBac1 vector were expressed in Sf9 cells infected with recombinant baculovirus (Bac-to-Bac, Invitrogen). Ni-NTA resin (Qiagen) was used to purify His_6_-tagged STIM1 for construction of STIM1 homodimers. To make heterodimers, His_6_-tagged and MBP-tagged STIM1 were coexpressed and purified in two steps by Ni-NTA followed by amylose resin (New England Biolabs).

### Protein Labeling and Reconstitution in Liposomes.

Sites for cysteine substitution and dye labeling were selected from outward-facing residues if a structure was available (EF-SAM, CAD), and if not, from residues predicted to lie outside interface surfaces. STIM1 dimer containing two cysteines was labeled with maleimide-conjugated Alexa Fluor 555 (donor dye) and Alexa Fluor 647 (acceptor dye; ThermoFisher). Liposomes were prepared from lipid mixtures extruded through 100-nm pores 30 times followed by addition of n-octyl-β-D-glucopyranoside (βOG). After addition of STIM1 protein, detergent was removed with Bio-Beads (Bio-Rad), and reconstituted proteoliposomes were purified with a Sepharose CL-4B column (Sigma Aldrich).

### Imaging Chamber Preparation.

Imaging chambers for single-molecule fluorescence imaging were prepared as described previously (30).

### TIRF Microscopy and smFRET Measurements.

All smFRET experiments were performed at room temperature on a home-built TIRF system based on an Axiovert S100 TV microscope equipped with a Fluar 100x 1.45 NA oil-immersion objective (Zeiss). 532- and 637-nm lasers (OBIS 532 nm LS 150 mW, Coherent, and OBIS 637 nm LX 140 mW, Coherent) were used for excitation in objective TIRF mode, and donor and acceptor emissions were collected with 580/60 nm and 731/137 nm bandpass filters (Semrock), respectively. Data were analyzed using custom Python scripts as detailed previously (30). The FRET ratio E was calculated at each time point as E = I_A_/(I_A_ + γI_D_), where I_A_ and I_D_ are the acceptor and donor fluorescence values, respectively, and γ was measured empirically for each molecule as described (30). Distances (R) were calculated from FRET (E) using the relation E = 1/[1 + (R/R_0_)^6^]. R_0_ for the dye pair was measured empirically on our smFRET system and had a value of 5.8 nm.

### Dye Position Simulation with Crystallography and NMR System (CNS).

We simulated dye positions on STIM1 structures using Crystallography and NMR System (CNS) as described (48).

### Generating Structural Models with AlphaFold2.

The ColabFold implementation of AlphaFold2 (49, 50) was used to generate models for residues 35 to 444 of human STIM1, with the following parameters: Num_relax = 0; Template_mode = none; MSA mode = mmseqs2_uniref_env; Pair_mode = unpaired_paired; Model_type = auto; Num_recycles = 48; Recycle_early_stop_tolerance = auto; Relax_max_iterations = 200; Pairing_strategy = greedy. Out of the top five models shown in *SI Appendix*, Fig. S3, only model 2 shows domain-swap binding between CC1 and CAD as well as overall agreement with smFRET-derived distances. This model was then processed using the Minimize Structure tool in Chimera (51) to eliminate clashes.

### Ca^2+^ Imaging.

Ca^2+^ imaging was conducted as described previously (30) using HEK293 cells cotransfected with Orai1-GFP and either WT or mutant mCherry-flSTIM1. Cells were loaded with 1 µM fura-2/AM (Invitrogen) in culture medium for 30 min at room temperature, washed, and plated on poly-D-lysine-treated coverslip chambers.

### Cysteine Crosslinking and Western Blot Analysis.

For crosslinking flSTIM1 in situ, STIM1/2 double knockout (DKO) HEK293 cells (52) were cotransfected with mCh-STIM1 and HA-STIM1 constructs with selected residues replaced by cysteine. 48 h after transfection, cells were exposed to 0.2 mM diamide for 10 min in Ringer’s solution containing 2 mM Ca^2+^. Cells were then lysed in RIPA buffer containing 20 mM NEM, 0.5 mM EDTA, and protease inhibitor cocktail (Cell Signaling Technology). Samples were run on SDS-PAGE and analyzed by Western blot using mouse anti-HA antibody (1:2,000, Sigma-Aldrich) and rabbit anti-mCherry antibody (1:2,000, OriGene Technologies), with secondary antibodies (680RD anti-mouse and 800CW anti-rabbit) on a LI-COR Odyssey imaging system.

Western blots were scanned and analyzed using ImageJ, and the area under each peak indicated the amount of protein in each band. Crosslinking efficiencies were calculated by dividing the amount of STIM1 in the mCherry x HA heterodimer band (crosslinked) by an estimate of the total amount of STIM1 heterodimer present in each sample (crosslinked + noncrosslinked). The intensities of the HA and mCherry channels were normalized using the mCherry x HA heterodimer band, which contains the same amount of STIM1 in both channels. The total amount of heterodimer was estimated from the total amounts of STIM1 in the mCherry and HA channels, assuming random assembly of the two subunits of the dimer.

## Supplementary Material

Appendix 01 (PDF)

## Data Availability

A PDB file for the AlphaFold2 model of STIM1 and original scans of Western blots related to Fig. 3 and *SI Appendix*, Fig. S4 have been deposited at Zenodo at https://doi.org/10.5281/zenodo.17715801 (53). All other data are included in the article and/or *SI Appendix*.
