# Supplementary material for: Multiple weak brakes act in concert to control STIM1 and store-operated calcium entry
Source: Proc Natl Acad Sci U S A. 2025 Dec 8;122(50):e2518622122. doi: 10.1073/pnas.2518622122 (PMC12718381; doi:10.1073/pnas.2518622122)
Supplement: Supplementary file 1 — Appendix 01 (PDF) [file pnas.2518622122.sapp.pdf]

Supporting information for

Multiple weak brakes act in concert to control STIM1 and store-operated calcium entry

Ruoyi Qiu and Richard S. Lewis

Department of Molecular and Cellular Physiology, Stanford University School of Medicine, Stanford, CA 94305

SI METHODS

DNA constructs. For insect cell expression, STIM1 constructs were cloned in the pFastBac1 vector by PCR from full-length human STIM1 (Origene) as follows. To construct his_6-STIM1, STIM1 residues 1-34 followed by a C-terminal his_6-tag and 3C protease cleavage sequence (LEVLFQGP) were inserted between EcoRI and XhoI restriction sites, and STIM1 residues 35-685 were inserted between XhoI and KpnI restriction sites. To construct MBP-STIM1, STIM1 residues 1-34 were inserted between BamHI and EcoRI restriction sites, the MBP sequence with a C-terminal 3C protease cleavage sequence was inserted between EcoRI and XhoI restriction sites, and STIM1 residues 35-685 were inserted between XhoI and KpnI restriction sites.

All native cysteines in STIM1 (C49, C56, C227 and C437) were mutated to serines, and desired mutations were introduced by site-directed mutagenesis (Quikchange XL; Stratagene). The activity of the cysteineless flSTIM1 appeared to be normal as assessed by resting $[Ca^{2+}]_i$ and thapsigargin-induced SOCE in HEK 293 cells cotransfected with Orai1 (**Fig. S1**). This result is consistent with normal puncta formation by a C227S/C437S STIM1 mutant after TG treatment (1) but differs from another study in which a single C437S mutation was reported to inhibit STIM1 activation (2). The reasons for this discrepancy are unclear.

His_6-TM-CT was made by replacing residues 35-685 of his_6-STIM1 with STIM1 residues 208-685. His_6-MBP-TM-CT was made by first linking MBP and 201-685 of STIM1 using Gibson assembly kit (New England Biolabs, E2611L) and substituting for residues 35-685 of his_6-STIM1.

For Ca^{2+} imaging in HEK 293 cells (**Fig. 5, Fig. S6)**, the cysteineless STIM1 and other STIM1 mutants were generated from mCherry-STIM1 (3) by site-directed mutagenesis (Quikchange XL; Stratagene). For cysteine crosslinking experiments in HEK 293 cells (**Fig. 3, Fig. S5**), constructs were generated from mCherry-STIM1 and HA-STIM1 (3) by site-

directed mutagenesis (Quikchange XL; Stratagene). C437, the only cysteine in the cytosolic domain, was mutated to serine and the desired residues were mutated to cysteine for diamide-induced crosslinking.

All plasmids were verified by Sanger sequencing or whole plasmid sequencing.

Protein expression and purification. All STIM1 constructs in pFastBac1 vector were expressed in Sf9 cells infected with recombinant baculovirus (Bac-to-Bac, Invitrogen). For symmetric inter-subunit smFRET samples, baculovirus of His_6-tagged STIM1 with a single cysteine substitution was used for expression. For asymmetric inter-subunit and intra-subunit smFRET samples, baculoviruses of His_6-tagged STIM1 and MBP-tagged STIM1 were used together for heterodimer expression.

Sf9 cells were harvested 45 h post infection and were lysed in a buffer of 20 mM Tris pH 7.8, 0.5 mM EDTA, 500 µM TCEP with added protease inhibitor cocktail (Sigma-Aldrich, S8830). Cell membranes were centrifuged at 150,000 × g for 30 min at 4°C. STIM1 protein was extracted from the membranes using a buffer of 20 mM Tris pH 7.2, 150 mM NaCl, 2% n-dodecyl-β-D-maltoside (DDM), 10 mM imidazole, 500 µM tris(2-carboxyethyl)phosphine (TCEP) and benzonase (1 µl/100 ml; Sigma-Aldrich) for 1 h at 4°C. After centrifugation, Ni-NTA resin (Qiagen) was added to the supernatant and rotated for 2 h at 4°C. Ni-NTA resin was washed with 20x column volume of wash buffer containing 20 mM Tris pH 7.2, 150 mM NaCl, 0.1% DDM, 500 µM TCEP, and 40 mM imidazole. Protein was eluted in wash buffer supplemented with 300 mM imidazole. To isolate heterodimers, amylose resin (New England Biolabs) was added to the elution and rotated for 1 h at 4°C, and protein was eluted in 20 mM Tris pH 7.2, 150 mM NaCl, 0.1% DDM, 500 µM TCEP, and 10 mM maltose. To cleave the tag, 3C protease was added to the eluted protein for 1 h at room temperature. Complete cleavage was verified by SDS-PAGE. After concentrating with a 100 kDa cutoff concentrator (Amicon Ultra), the STIM1 protein was further purified by size exclusion chromatography (SEC) using a Superose 6

Increase 10/300 GL column (Cytiva) equilibrated with SEC buffer of 20 mM Tris pH 7.2, 150 mM NaCl, 0.1% DDM, and 100 µM TCEP. Fractions containing STIM1 protein were collected and concentrated with a 100 kDa cutoff concentrator (Amicon Ultra). The STIM1 protein was supplemented with 10% (v/v) glycerol and flash frozen in liquid nitrogen. The purity of STIM1 samples were higher than 90% as determined by SDS-PAGE.

Protein labeling and reconstitution in liposomes. Sites for cysteine substitution and dye labeling were selected from outward-facing residues if a structure was available (EF-SAM, CAD), and if not, from residues predicted to lie outside interface surfaces. STIM1 dimer containing two cysteines was labeled with maleimide-conjugated Alexa Fluor 555 and Alexa Fluor 647 (ThermoFisher). STIM1 was diluted to 10 µM in 200 µl desalting buffer (20 mM Tris pH 7.2, 150 mM NaCl, 0.1% DDM, and 100 µM TCEP). 5 µM donor fluorophore and 5 µM acceptor fluorophore were added and after incubation for 1 h at 4°C, free dye was removed with a home-packed desalting column filled with 5 ml of G50 resin (Sigma) pre-equilibrated with desalting buffer. Labeling efficiency was ~50% for most samples. The labeled STIM1 protein was concentrated to ~10 µM, aliquoted, supplemented with 10% (v/v) glycerol and flash frozen.

To reconstitute STIM1 proteins in liposomes, egg PC, POPE, and POPG (Avanti) were mixed at a 3:1:1 ratio in chloroform, and 0.5% biotinylated PE (Avanti) was added. In some experiments (Fig. 4B, Fig. S5A), egg PC and either DOPS (Avanti) or POPE were mixed in a 4:1 ratio. The lipids in chloroform were dried by nitrogen gas and kept in vacuum overnight. The dry lipids were resuspended in buffer containing 20 mM Tris pH 7.2 and 150 mM NaCl to a final concentration of 10 mg/ml, creating a cloudy solution. Using an extruder set (Avanti Polar Lipids) the lipids were then extruded through a membrane with 100-nm pores (Whatman) 30 times to form liposomes with a diameter of ~100 nm. For each sample, 180 µl of liposomes (10 mg/ml) was added to 20 µl of 12% n-octyl-β-D-glucopyranoside (βOG) solubilized in the same buffer and rotated for 15

min at 4°C. 10 µl of labeled STIM1 protein (~10 µM) was added to the liposomes and rotated for 30 min at 4°C. 25 mg of Bio-Beads (Bio-Rad) was added to the sample and rotated for 1 h at 4°C, and another 25 mg of Bio-Beads was added and rotated for 1 additional hour. The reconstituted proteoliposomes were separated from free detergent and free STIM1 protein using a home-packed Sepharose CL-4B column (Sigma Aldrich). The fraction containing STIM1 proteoliposomes was added to 10 mg Bio-Beads and rotated overnight at 4°C to remove residual detergent, at which point the sample was ready for smFRET experiments.

Imaging chamber preparation. Imaging chambers for single-molecule fluorescence imaging were prepared according to established protocols (4). Briefly, microscope slides and coverslips were first cleaned by stepwise sonication for 15 min each in glass containers with acetone, ethanol, 1 M KOH and pure water. They were then coated with a 100:1 PEG/PEG-biotin mixture (Laysan Bio) prior to flow cell construction. Strips of double-sided tape were applied to a quartz microscope slide (Finkenbeiner) to form channel walls, with holes at both ends of each channel to allow exchange of sample solutions. A microscope coverslip of 1.5 thickness (Erie Scientific) was pressed on the tape strips and edges of the channels were sealed with epoxy glue (Devcon).

Prior to attaching biotinylated liposomes to the coverslip surface, channels were rinsed with 20/150 TBS (150 mM NaCl, 20 mM Tris, pH 7.2 with HCl) then incubated with 0.2 mg/ml neutravidin (Thermo Fisher CWA) for 5 min. Liposomes were loaded into the channels after washing out the neutravidin. Prior to TIRF imaging, channels were filled with 20/150 TBS containing 100 µM cyclooctatetraene (Sigma Aldrich) and an oxygen scavenging system consisting of 1 % D-glucose, 1 mg/ml glucose oxidase (Sigma Aldrich), and 0.04 mg/ml catalase (Sigma Aldrich).

TIRF microscopy and smFRET measurements. All smFRET experiments were performed at room temperature following a previous protocol (5) with some modifications. The

home-built TIRF system is based on an Axiovert S100 TV microscope equipped with a Fluar 100x 1.45 NA oil-immersion objective (Zeiss). 532- and 637-nm lasers (OBIS 532 nm LS 150 mW, Coherent and OBIS 637 nm LX 140 mW, Coherent) were used for excitation in objective TIRF mode. Donor and acceptor signals were separated by a 652 nm dichroic (Semrock) and passed through 580/60 nm and 731/137 nm bandpass filters (Semrock), respectively, mounted in an OptoSplit-II beamsplitter (Cairn Research) to an EM-CCD camera (iXon DU897E, Andor). Hardware and data acquisition were controlled by homemade scripts in µManager as described (5).

For each sample, the molecule density and distribution on the coverslip surface was checked with the TIRF microscope. When optimal density was achieved (300 to 500 molecules in the camera field of view), donor and acceptor emission were recorded with a 100-ms integration time under 532-nm laser excitation for 80 s, immediately followed by 1 s of 637-nm laser excitation to verify acceptor dye bleaching.

Data were analyzed using custom Python scripts as detailed previously (5). Briefly, molecules with single acceptor bleaching before single donor bleaching were selected. The FRET ratio E was calculated at each time point as $E = I_A/(I_A + \gamma I_D)$, where I_A and I_D are the acceptor and donor fluorescence values, respectively, and γ was measured empirically for each molecule as described previously (5). For single-molecule traces longer than 20 points (2 s), summed FRET histograms were constructed by distributing FRET amplitudes into 40 bins (from –0.25 to 1.25) and normalizing by the number of points in each trace. Histograms were fitted with a sum of Gaussian functions using routines written in Igor Pro (WaveMetrics).

R_0 of the dye pair was measured empirically on our smFRET system. The crystal structure of CAD was used to simulate the dye-dye distances for 417-417’ and 431-431’ pairs using Crystallography and NMR System (CNS) (6). These distances were used to solve for R_0 using the FRET equation and 417:417’ and 431:431’ smFRET measurements

from ctSTIM1 samples. The calculated R_0 values were 5.85 nm for 417:417′ and 5.80 nm for 431:431′; a value of 5.8 nm was used to estimate distances throughout this paper. Distances (R) were calculated from FRET (E) using the relation $E = 1/[1 + (R/R_0)^6]$.

Dye position simulation with Crystallography and NMR System (CNS). We simulated dye positions on STIM1 structures using Crystallography and NMR System (CNS) as described (7). Briefly, the pdb file was loaded in Pymol and residues for dye labeling were mutated to cysteine. Atomic models of the fluorophores and their maleimide linker were attached to the mutated cysteines and subjected to molecular dynamics simulations by fixing all protein atoms except the dye and linker atoms. 100 simulations were performed for each labeling pair and the average distance between the dye centers (CAO atom) was calculated from the resulting coordinates.

Generating structural models with AlphaFold2. The ColabFold implementation of AlphaFold2 (8, 9) was used to generate models for residues 35-444 of human STIM1, with the following parameters: Num_relax = 0; Template_mode = none; MSA mode = mmseqs2_uniref_env; Pair_mode = unpaired_paired; Model_type = auto; Num_recycles = 48; Recycle_early_stop_tolerance = auto; Relax_max_iterations = 200; Pairing_strategy = greedy. Out of the top five models shown in **Fig. S3**, only model 2 shows domain-swap binding between CC1 and CAD as well as overall agreement with smFRET-derived distances. This model was then processed using the Minimize Structure tool in Chimera (10) to eliminate clashes.

In all structures, only residues from the cEF hand to the N-terminal end of CAD (63-444) are shown. Residues beyond 444 are predicted to be unstructured and in our initial AlphaFold2 modeling of STIM1 35-685 showed low confidence (pIDDT values <0.5). While the model accurately predicted inter-dye distances estimated from smFRET in the cytosolic region, it needs refinement for the TM and luminal domains. The model underestimated the TM spacing (222:222′; smFRET distance 6.4 nm, model distance 4.1

nm), which may be related to the fact that AlphaFold2 generates a continuous helix between TM and CC1α1 (aa 233-236) rather than a non-helical segment as predicted by JPRED3 (11), possibly resulting in a rotation of the TM helices that orients the 222 residue sidechains to face each other. In addition, while the cEF hand-SAM distance (64:178) from smFRET (4.1 nm) was consistent with dye positions simulated with the published EF-SAM NMR structure (4.1 nm), it differed from the AlphaFold2 model (2.0 nm). For these reasons, we only applied the model to make predictions about the cytosolic region.

Ca^{2+} imaging. HEK293 cells were co-transfected with WT or mutant mCherry-flSTIM1 and Orai1-GFP. 24–28 h after transfection, cells were loaded with 1 µM fura-2/AM (Invitrogen) in culture medium for 30 min at room temperature, washed, and plated on poly-D-lysine-treated coverslip chambers. Ca^{2+} imaging was conducted as described previously (5) using a Zeiss 200M inverted microscope equipped with a Fluar 40X NA 1.3 oil-immersion objective. Cells were excited alternately at 350 and 380 nm (Polychrome II, TILL Photonics), and emission at 534 ± 30 nm (Semrock) was collected with a Flash4.0 sCMOS camera (Hamamatsu Corp.) with 2 × 2 binning. Images were background-corrected before calculating the mean 350/380 ratio for each cell. Standard 2 Ca Ringer's solution contained (in mM): 155 NaCl, 4.5 KCl, 2 $CaCl_2$, 1 $MgCl_2$, 10 D-glucose and 5 Na-HEPES (pH 7.4). Ca^{2+}-free (0 Ca) Ringer's was prepared by replacing $CaCl_2$ with 2 mM $MgCl_2$ and 1 mM EGTA. Thapsigargin (Sigma-Aldrich) was diluted from a 1 mM stock in DMSO in Ca^{2+}-free Ringer's.

SUPPLEMENTARY FIGURES

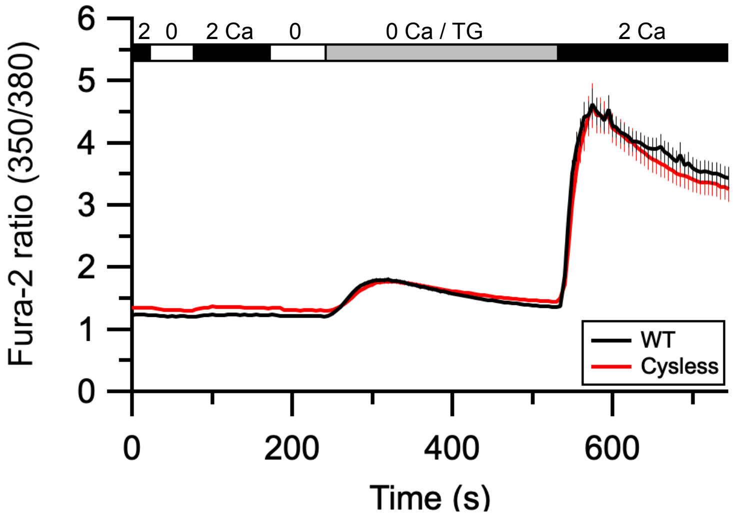

Figure S1. Comparison of SOCE evoked by WT STIM1 vs cysteineless STIM1. STIM1/2 DKO HEK293 cells were cotransfected with Orai1 and either WT mCh-STIM1 or mCh-STIM1 with all endogenous cysteines (C49, C56, C227, and C437) replaced by serines ('cysless'). Fura-2 350/380 ratios are shown (mean ± sem; WT, from Fig. 5C, n=57 cells; cysless, n=54 cells). Solution changes are shown with [Ca^{2+}] in mM and 1 µM TG added as indicated to deplete Ca^{2+} stores. Cysless STIM1 is fully functional as assessed by SOCE observed upon Ca^{2+} readdition.

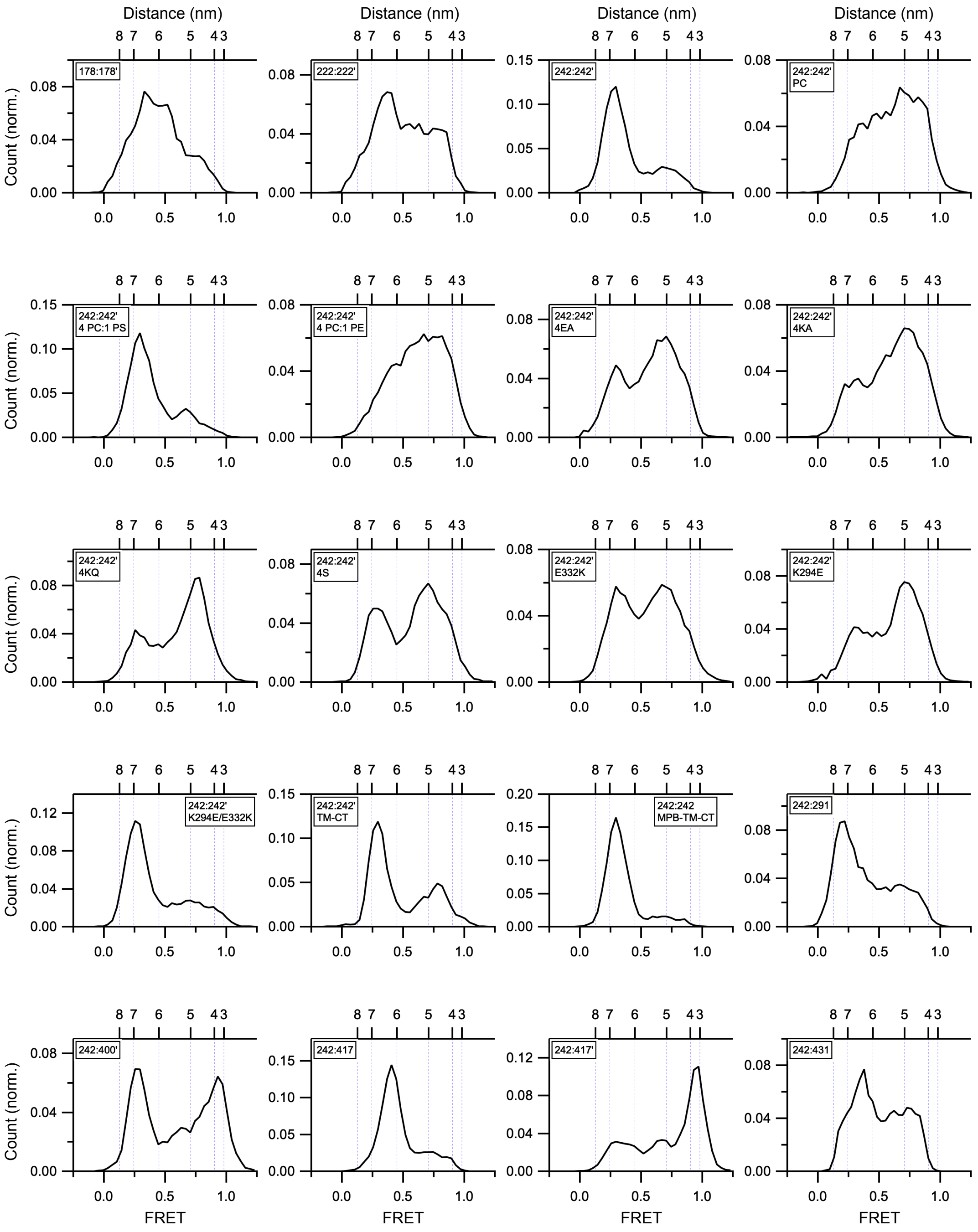

Distance (nm)
Count (norm.)
FRET
178:178'
222:222'
242:242'
242:242' PC
242:242' 4 PC:1 PS
242:242' 4 PC:1 PE
242:242' 4EA
242:242' 4KA
242:242' 4KQ
242:242' 4S
242:242' E332K
242:242' K294E
242:242' K294E/E332K
242:242' TM-CT
242:242 MPB-TM-CT
242:291
242:400'
242:417
242:417'
242:431

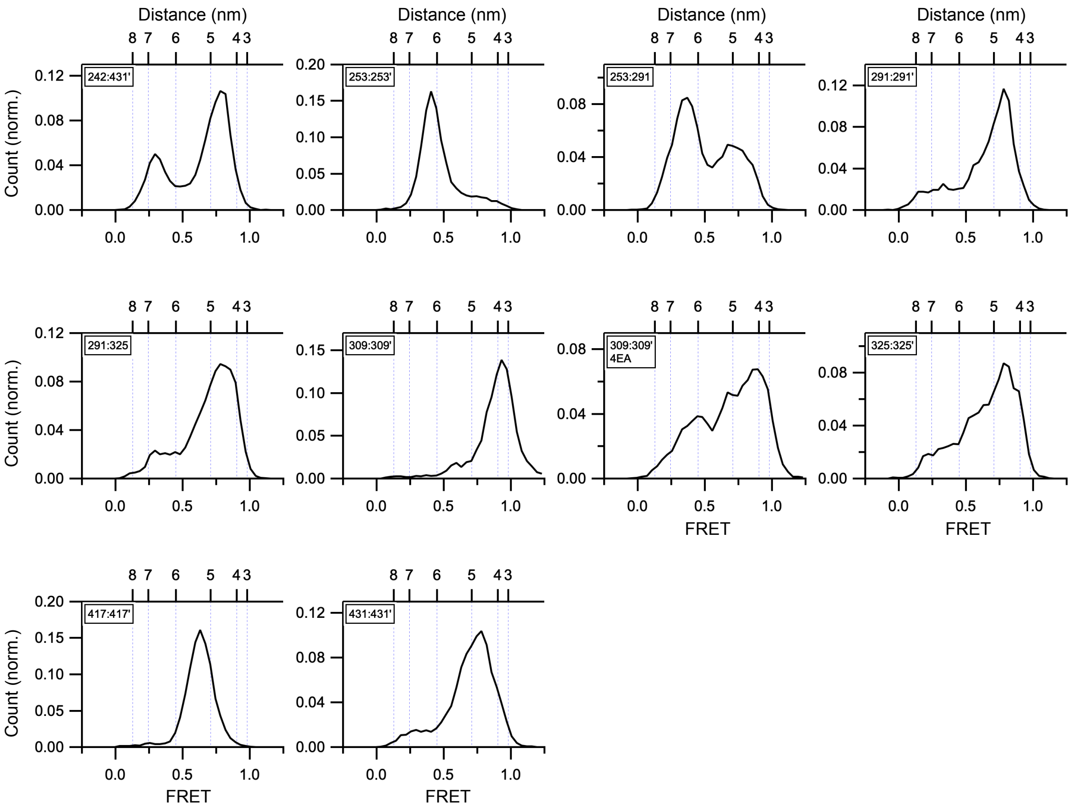

Figure S2. smFRET amplitude histograms for flSTIM1 in 2 mM Ca^{2+}. Predominant peak FRET values and number of molecules used to construct the histogram are listed in Table S1. Abbreviations: PC (phosphatidylcholine liposomes), 4 PC : 1 PS (4:1 PC and phosphatidylserine liposomes), 4 PC : 1 PE (4:1 PC and phosphatidylethanolamine liposomes), 4EA (E318A/E319A/E320A/E322A), 4KA (K382A/K384A/K385A/K386A), 4KQ (K382Q/K384Q/K385Q/K386Q), 4S (L321S/V324S/L328S/L335S), TM-CT (STIM1 208-685), MBP-TM-CT (MBP+STIM1 201-685).

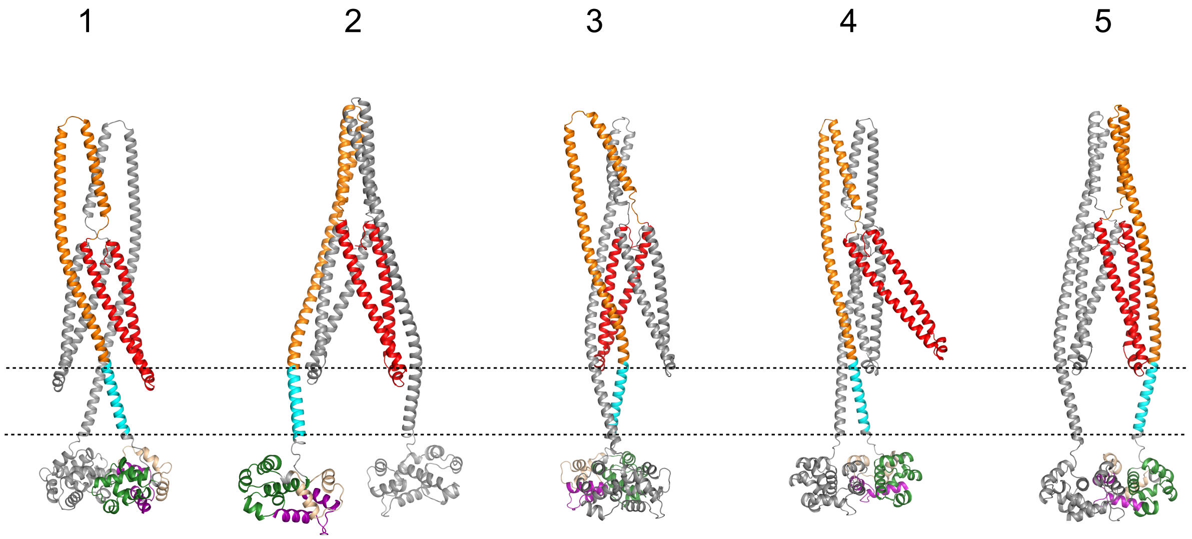

Figure S3. AlphaFold2 models of flSTIM1. Top five models generated by AlphaFold2, using STIM1 residues 35-444 as input. For each model, the regions from the cEF hand through CAD (63-444) are shown. Domains are colored as shown in Fig. 1, and predicted membrane edges based on the TM domain are shown by dashed lines. Only model 2 is consistent with distances derived from smFRET.

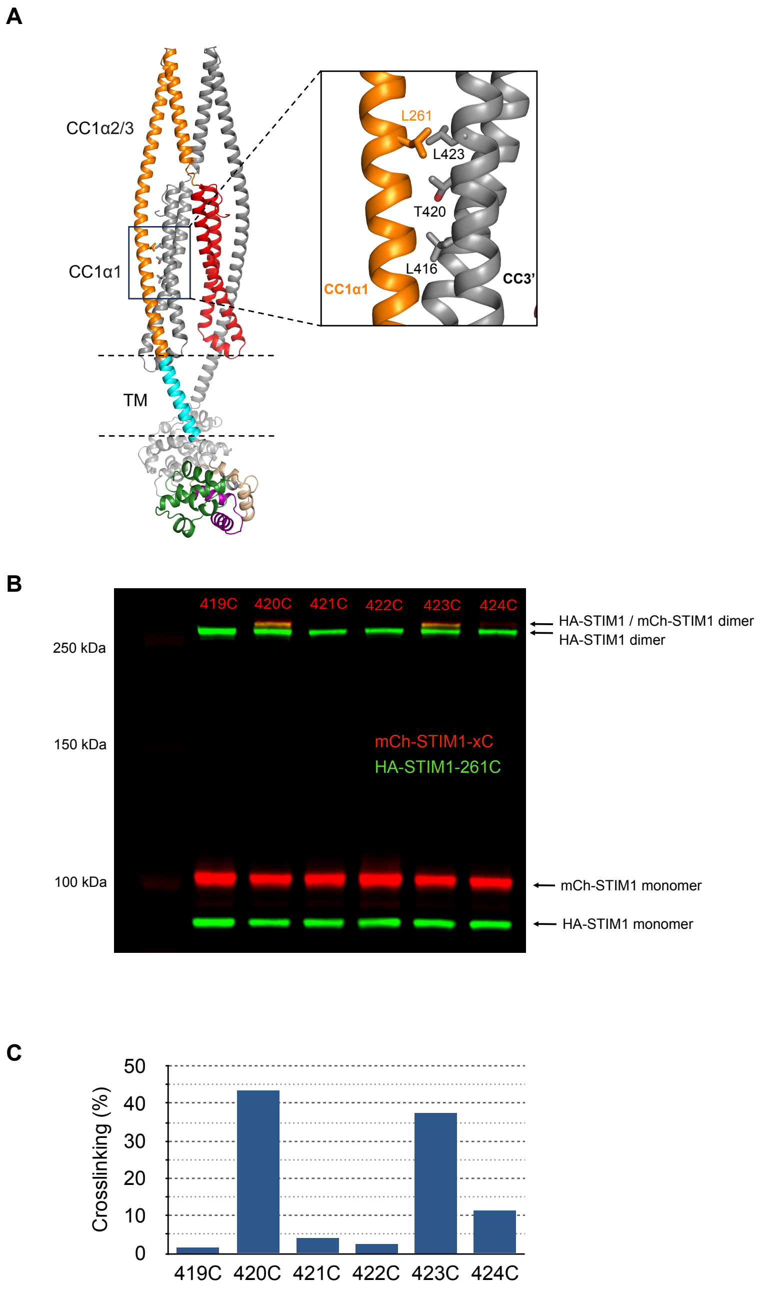

Figure S4. Helical alignment of L261C in the CC1α1-CC3 brake. (*A*) Proximity of L261 (CC1α1) and T420 and L423 (CC3) predicted by the AlphaFold2 model. (*B*) Western blot showing heterodimer disulfide crosslinking between HA-STIM1-L261C and mCh-STIM1-T420C and -L423C. Each lane contains the lysate from diamide-treated cells coexpressing HA-STIM1-L261C and a single mCherry-STIM1 mutant (red) (see Methods). (*C*) Percent of heterodimers forming disulfide crosslinks, measured from the gel in *B* (see Methods). Results are representative of two experiments.

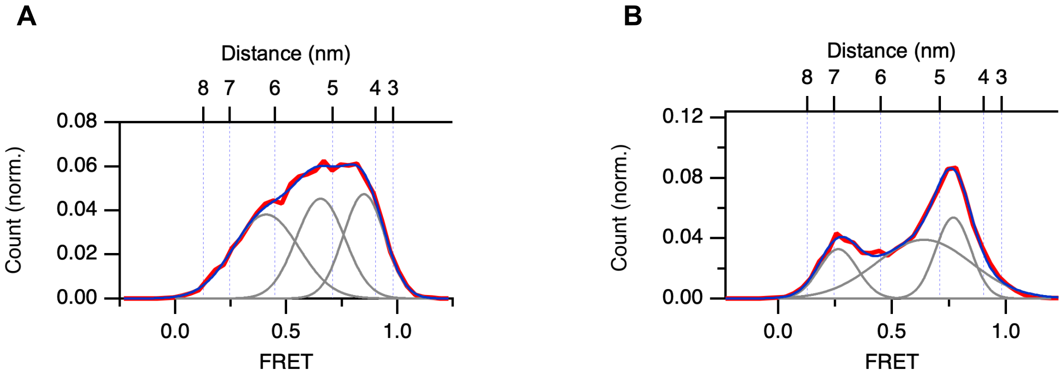

Figure S5. Effects of PC/PE liposomes and 4KQ mutation on the stability of the resting STIM1 conformation. (*A*) smFRET histogram for 242:242’ of flSTIM1 reconstituted in liposomes containing 4 PC:1 PE (uncharged; n=220) in 2 mM Ca^{2+}. Fitted Gaussian curves (*gray*) and their sum (*blue*) are superimposed on the data (*red*). Fit parameters (peak FRET and fractional area): 0.41 (38%), 0.66 (33%), 0.85 (29%). (*B*) smFRET histogram for 242:242’ of flSTIM1-4KQ (n=207) reconstituted in standard liposomes containing 3 PC:1 PE:1 PG (20% negative charge; n=207) in 2 mM Ca^{2+}. Fit parameters (peak FRET and fractional area): 0.26 (18%), 0.64 (54%), 0.77 (28%).

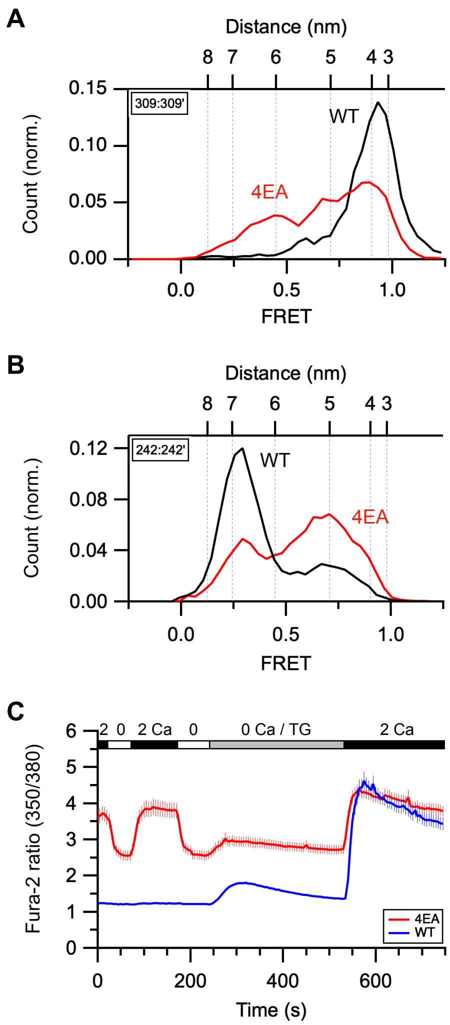

Figure S6. Effects of the 4EA mutation on stability of the resting STIM1 conformation. (*A*) smFRET histograms of 309:309' for STIM1-WT (n=76) and STIM1-4EA (E318A/E319A/E320A/E322A; n=225) in 2 mM Ca^{2+}. (*B*) smFRET histograms of 242:242' for STIM1-WT (n=361) and STIM1-4EA (n=225) in 2 mM Ca^{2+}. (*C*) Fura-2 350/380 ratios (mean ± sem) from HEK cells expressing Orai1 and either STIM1-WT (from Fig. 5*C*, n=57 cells), or STIM1-4EA (n=62 cells). Changes in extracellular [Ca^{2+}] (0 and 2 mM) and addition of thapsigargin (TG; 1 µM) are indicated. Resting $[Ca^{2+}]_i$ is elevated in cells expressing STIM1-4EA, indicating constitutive activity of STIM1 and SOCE, while TG-induced SOCE is normal.

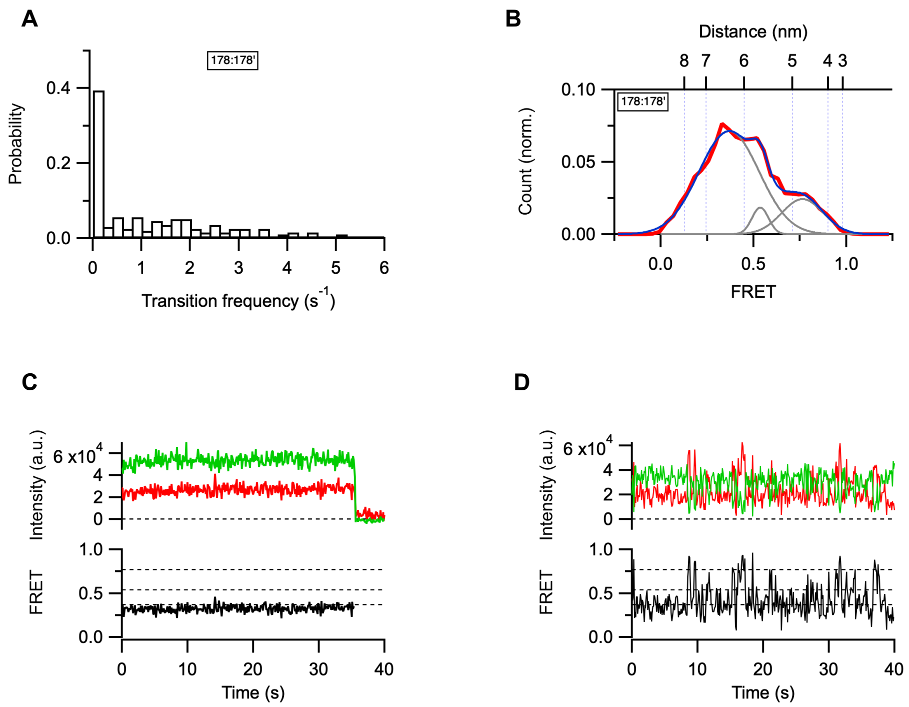

Figure S7. Conformational dynamics of flSTIM1 luminal SAM domains. (*A*) Probability distribution of the mean smFRET transition frequency over a 5-s period for the 178:178' dye pair (SAM-SAM; n=271). (*B-D*) smFRET histogram for 178:178' (*B*) and representative single molecule traces showing stable (*C*) and fluctuating (*D*) molecules. Dashed lines indicate peak values from the smFRET histogram. Fit parameters (peak FRET and fractional area): 0.37 (77%), 0.54 (5%), 0.77 (18%).

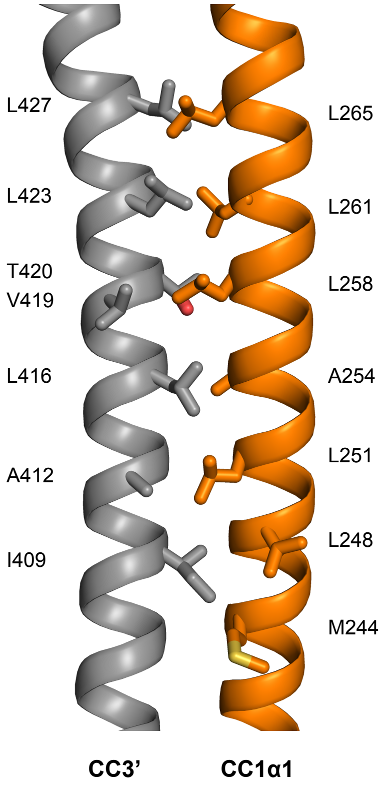

Figure S8. The CC1α1-CC3 hydrophobic interface. Domain-swapped CC1α1 and CC3' helices show multiple hydrophobic contacts between residues identified by mutagenesis as critical for maintaining the CC1 clamp (see Table S3). The CC2' helix has been omitted for clarity.

Table S1. Summary of smFRET measurements for the resting state of flSTIM1 in saturating (2 mM) Ca^{2+}

Dye pair	Peak FRET	Number of molecules	Distance from FRET (nm)	Distance from AlphaFold2 (nm)
178:178'	0.33	271	6.53	11.69
222:222'	0.33	114	6.53	3.96
242:242'	0.28	361	6.81	7.36
242:291	0.22	192	7.16	8.02
242:400'	0.93	188	3.77	3.76
242:417	0.41	291	6.16	6.69
242:417'	0.97	190	3.27	2.86
242:431	0.35	298	6.43	7.57
242:431'	0.80	189	4.60	4.13
253:253'	0.41	281	6.18	6.35
253:291	0.37	254	6.34	6.27
291:291'	0.78	185	4.70	4.70
291:325	0.78	202	4.70	3.89
309:309'	0.93	76	3.76	3.03
325:325'	0.80	243	4.60	3.63
417:417'	0.63	169	5.31	5.09
431:431'	0.78	204	4.70	4.85

For each dye pair, the predominant smFRET value from the STIM1-WT amplitude histograms is listed and used to calculate a distance. The number of single molecule traces used to construct each histogram is indicated. Inter-dye distances from the AlphaFold2 model were measured as described in SI Appendix, Methods.

Table S2. Comparison of smFRET measurements in flSTIM1 vs ctSTIM1

Dye pair	flSTIM1	ctSTIM1
242:242′	0.29	0.31
242:400′	0.93	0.95
242:417	0.41	0.40
242:417′	0.97	0.90
242:431	0.35	0.30
242:431′	0.80	0.73
309:309′	0.93	0.90
417:417′	0.63	0.64
431:431′	0.78	0.77

The table lists the predominant smFRET values for each indicated dye pair. ctSTIM1 values are reproduced from our previous study (5).

Table S3. Interactions of critical residues in the CC1 clamp predicted by the AlphaFold2 model

CC1α1	CC2'	CC3'	Mutations and references
M244		I409'	M244A (13), M244S (14)<br><br>I409A (13), I409S (14)
L248		I409'	L248A (13), L248S (15)<br><br>I409A (13), I409S (14)
L251	L373'	I409'<br><br>L416'	L251A (13), L251S (15–18)<br><br>L373A (13)<br><br>I409A (13), I409S (14)<br><br>L416A (13), L416S-L423S (15), L416G/A (18)
L258		V419'	L258A (13), L258S (15), L258G/A (18)<br><br>V419A (13), V419G (18)
L261		T420'<br><br>L423'	L261G (18)<br><br>T420K (14)<br><br>L423A (13), L423G (18), L416S-L423S (15)
L265		L423'<br><br>L427'	L265A (13)<br><br>L423A (13), L423G (18), L416S-L423S (15)<br><br>L427A (13)

The table lists residues that were identified by mutagenesis to be essential for stabilizing the CC1 clamp. Residues in red destabilize the clamp based on increased CC1-CAD binding when substituted by alanine. The interface is pictured in Fig. S8.

REFERENCES

1. N. Hirve, V. Rajanikanth, P. G. Hogan, A. Gudlur, Coiled-coil formation conveys a STIM1 signal from ER lumen to cytoplasm. *Cell Rep.* **22**, 72–83 (2018).

2. G. Kodakandla, *et al.*, Dynamic S-acylation of the ER-resident protein stromal interaction molecule 1 (STIM1) is required for store-operated Ca^{2+} entry. *J. Biol. Chem.* **298**, 102303 (2022).

3. M. M. Wu, J. Buchanan, R. M. Luik, R. S. Lewis, Ca^{2+} store depletion causes STIM1 to accumulate in ER regions closely associated with the plasma membrane. *J. Cell Biol.* **174**, 803–813 (2006).

4. R. Roy, S. Hohng, T. Ha, A practical guide to single-molecule FRET. *Nat. Methods* **5**, 507–516 (2008).

5. S. van Dorp, *et al.*, Conformational dynamics of auto-inhibition in the ER calcium sensor STIM1. *Elife* **10**, e66194 (2021).

6. A. T. Brunger, Version 1.2 of the Crystallography and NMR system. *Nat. Protoc.* **2**, 2728–33 (2007).

7. U. B. Choi, *et al.*, Single-molecule FRET-derived model of the synaptotagmin 1-SNARE fusion complex. *Nat. Struct. Mol. Biol.* **17**, 318–24 (2010).

8. J. Jumper, *et al.*, Highly accurate protein structure prediction with AlphaFold. *Nature* **596**, 583–589 (2021).

9. M. Mirdita, *et al.*, ColabFold: making protein folding accessible to all. *Nat. Methods* **19**, 679–682 (2022).

10. E. F. Pettersen, *et al.*, UCSF Chimera - A visualization system for exploratory research and analysis. *J. Comput. Chem.* **25**, 1605–1612 (2004).

11. J. Soboloff, B. S. Rothberg, M. Madesh, D. L. Gill, STIM proteins: dynamic calcium signal transducers. *Nat. Rev. Mol. Cell Biol.* **13**, 549–565 (2012).

12. Y. Zhou, *et al.*, Cross-linking of Orai1 channels by STIM proteins. *Proc Natl Acad Sci U S A* **115**, E3398–E3407 (2018).

13. N. Shrestha, *et al.*, Mapping interactions between the CRAC activation domain and CC1 that regulate the activity of the ER Ca^{2+} sensor STIM1. *J. Biol. Chem.* 102157 (2022). https://doi.org/10.1016/j.jbc.2022.102157.

14. F. Horvath, *et al.*, Swing-out opening of stromal interaction molecule 1. *Protein Sci.* **32**, 1–17 (2023).

15. M. Muik, *et al.*, STIM1 couples to ORAI1 via an intramolecular transition into an

extended conformation. *EMBO J.* **30**, 1678–1689 (2011).

16. M. Fahrner, *et al.*, A Coiled-coil Clamp Controls Both Conformation and Clustering of Stromal Interaction Molecule 1 (STIM1). *J. Biol. Chem.* **289**, 33231–33244 (2014).

17. Y. Zhou, *et al.*, Initial activation of STIM1, the regulator of store-operated calcium entry. *Nat. Struct. Mol. Biol.* **20**, 973–981 (2013).

18. G. Ma, *et al.*, Inside-out Ca^{2+} signaling prompted by STIM1 conformational switch. *Nat. Commun.* **6**, 7826 (2015).
